# Epicardioid single-cell genomics uncovers principles of human epicardium biology in heart development and disease

**DOI:** 10.1038/s41587-023-01718-7

**Published:** 2023-04-03

**Authors:** Anna B. Meier, Dorota Zawada, Maria Teresa De Angelis, Laura D. Martens, Gianluca Santamaria, Sophie Zengerle, Monika Nowak-Imialek, Jessica Kornherr, Fangfang Zhang, Qinghai Tian, Cordula M. Wolf, Christian Kupatt, Makoto Sahara, Peter Lipp, Fabian J. Theis, Julien Gagneur, Alexander Goedel, Karl-Ludwig Laugwitz, Tatjana Dorn, Alessandra Moretti

**Affiliations:** 1 First Department of Medicine, Cardiology, Klinikum rechts der Isar, Technical University of Munich, School of Medicine and Health, Munich, Germany; 2 Regenerative Medicine in Cardiovascular Diseases, First Department of Medicine, Klinikum rechts der Isar, Technical University of Munich, School of Medicine and Health, Munich, Germany; 3 German Center for Cardiovascular Research (DZHK), Munich Heart Alliance, Munich, Germany; 4 Department of Experimental and Clinical Medicine, University ‘Magna Graecia’, Catanzaro, Italy; 5 School of Computation, Information and Technology, Technical University of Munich, Garching, Germany; 6 Computational Health Center, Helmholtz Center Munich, Neuherberg, Germany; 7 Helmholtz Association—Munich School for Data Science (MUDS), Munich, Germany; 8 Center for Molecular Signaling (PZMS), Institute for Molecular Cell Biology, Research Center for Molecular Imaging and Screening, Medical Faculty, Saarland University, Homburg, Germany; 9 Department of Congenital Heart Defects and Pediatric Cardiology, German Heart Center Munich, Technical University of Munich, School of Medicine and Health, Munich, Germany; 10 Department of Cell and Molecular Biology, Karolinska Institute, Stockholm, Sweden; 11 Department of Surgery, Yale University School of Medicine, New Haven, CT USA; 12 Institute of Human Genetics, School of Medicine, Technical University of Munich, Munich, Germany

**Keywords:** Pluripotent stem cells, Regenerative medicine, Induced pluripotent stem cells

## Abstract

The epicardium, the mesothelial envelope of the vertebrate heart, is the source of multiple cardiac cell lineages during embryonic development and provides signals that are essential to myocardial growth and repair. Here we generate self-organizing human pluripotent stem cell-derived epicardioids that display retinoic acid-dependent morphological, molecular and functional patterning of the epicardium and myocardium typical of the left ventricular wall. By combining lineage tracing, single-cell transcriptomics and chromatin accessibility profiling, we describe the specification and differentiation process of different cell lineages in epicardioids and draw comparisons to human fetal development at the transcriptional and morphological levels. We then use epicardioids to investigate the functional cross-talk between cardiac cell types, gaining new insights into the role of IGF2/IGF1R and NRP2 signaling in human cardiogenesis. Finally, we show that epicardioids mimic the multicellular pathogenesis of congenital or stress-induced hypertrophy and fibrotic remodeling. As such, epicardioids offer a unique testing ground of epicardial activity in heart development, disease and regeneration.

## Abstract (web summary)

The lineage of human epicardium is studied using time course single-cell analysis of epicardioids.

## Main

The epicardium is the mesothelial cell sheet covering the heart’s outer surface. Long considered a simple barrier between the pericardial cavity and the myocardium, it is now recognized to hold key functions in cardiac development and repair. During embryonic development, a subset of epicardial cells undergoes epithelial-to-mesenchymal transition (EMT) to become epicardial-derived cells (EPDCs) that migrate into the myocardium and give rise to the majority of fibroblasts and mural cells (vascular smooth muscle cells (SMCs) and pericytes) of the heart^1^. Whether EPDCs also differentiate into cardiomyocytes (CMs) and coronary endothelial cells is still debated, with studies providing conflicting evidence^1,2^. In addition to these cellular contributions, the epicardium provides signaling factors critical for the development and growth of the myocardium^3,4^ and also plays a central role in heart regeneration in species capable of rebuilding adult heart muscle after injury, such as zebrafish, making it a highly promising target for therapy^1^. However, the inaccessibility of human embryonic tissue at early stages of epicardium development, which begins less than 4 weeks after conception, has left substantial gaps in our understanding of human epicardial development and function. Many questions on the ontogeny of human epicardial precursors and the functional heterogeneity of epicardial cells are still unresolved, which limits harnessing their full potential for regenerative medicine.

Pluripotent stem cell (PSC)-derived cardiac organoids have emerged as powerful in vitro models of human development and disease^5,6^, but none have yet demonstrated the spontaneous formation of a bona fide epicardial compartment. Here, we generated cardiac organoids showing self-organization of highly functional ventricular myocardium and epicardium, which we called epicardioids. Time course single-cell genomics in epicardioids combined with lineage tracing revealed principles of human epicardial origin and biology, including the developmental trajectories of the epicardial lineage and the functional cross-talk with other cardiac cell types. In addition, we show that epicardioids represent an advanced system to model multicellular mechanisms of heart disease.

## Results

### Generation of epicardioids from human PSCs (hPSCs)

**Fig. 1:**
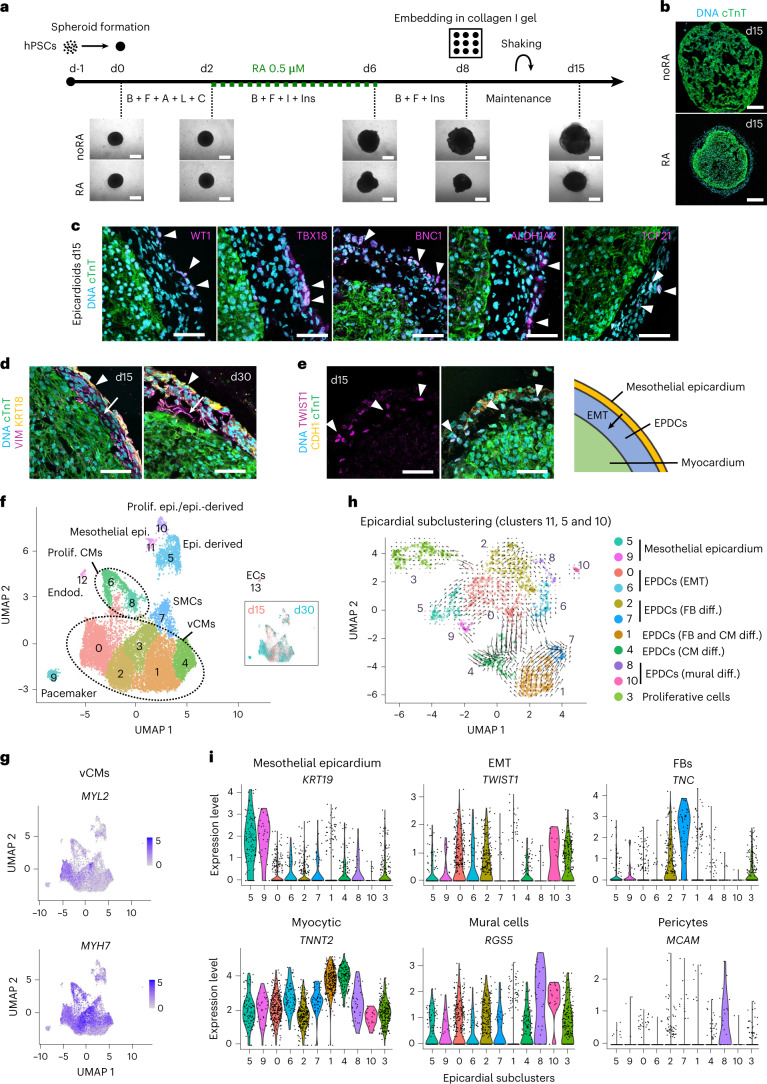
Generation of hPSC-derived epicardioids showing self-organized ventricular myocardium and epicardium. **a**, Top, protocol used for 3D cardiac induction of hPSCs, with or without the addition of RA. Bottom, representative brightfield images at the indicated days (d); B, BMP4; F, FGF2; A, activin A; L, LY-29004; C, CHIR-99021; I, IWP2; Ins, insulin; scale bars, 500 µm. **b**, Immunostaining for the CM marker cTnT in spheroids differentiated with or without RA (day 15 (d15)); scale bars, 200 µm. **c**, Immunostaining for cTnT and the epicardial markers WT1, TBX18, BNC1, ALDH1A2 and TCF21 in spheroids differentiated with RA, called epicardioids hereafter (day 15); scale bars, 50 µm. **d**, Immunostaining for cTnT, the mesenchymal marker vimentin (VIM) and the epithelial marker cytokeratin 18 (KRT18) in epicardioids at days 15 and 30. Arrowheads indicate the mesothelial epicardium, and arrows indicate subjacent EPDCs; scale bars, 50 µm. **e**, Left, immunostaining for cTnT, the epithelial marker E-cadherin (CDH1) and the EMT marker TWIST1 in epicardioids at day 15; scale bars, 50 µm. Right, schematic of the outer mesothelial epicardium layer and EPDCs arising through epicardial EMT in epicardioids. **f**, UMAP dimensional reduction plot showing the 14 cell clusters obtained by scRNA-seq of epicardioids at days 15 and 30; main cell types are annotated. The inset shows cells from day 15 and day 30 labeled in red and blue, respectively. **g**, Feature plots showing the expression levels of the vCM markers *MYH7* and *MYL2*. **h**, UMAP plot of the subclustering of the epicardial clusters 11, 5 and 10 overlaid with the trajectories inferred from RNA velocity; cell types are annotated. **i**, Violin plots showing the expression levels of markers of mesothelial epicardium (*KRT19*), EMT (*TWIST1*), fibroblasts (FBs; *TNC*), CMs (*TNNT2*), mural cells (*RGS5*) and pericytes (*MCAM*) in the epicardial subclusters presented in **h**; Prolif., proliferating, Epi., epicardium; Endod., endodermal cells; ECs, endothelial cells; diff., differentiation. Images in **a** and **b** and **c**–**e** are representative of six and three independent differentiations, respectively.

The formation of organoids relies on the self-patterning of cells following minimal stimulation of the signaling pathways that drive organ development in vivo. A key regulator of cardiac anteroposterior patterning is retinoic acid (RA), a metabolite of vitamin A that is also implicated in epicardial development^7,8^ and promotes the differentiation of hPSCs into epicardial cells in vitro^9–11^. To establish cardiac organoids containing an epicardial compartment, we generated hPSC spheroids in 96-well U-bottom plates and exposed them to a differentiation protocol driving the stepwise induction of the midanterior primitive streak, cardiac mesoderm and cardiovascular derivatives by modulation of Wnt, activin A, bone morphogenic protein 4 (BMP4) and basic fibroblast growth factor (bFGF) signaling, either with or without (noRA) the addition of 0.5 µM RA from days 2 to 5 (Fig. 1a)^12,13^. After the removal of differentiation cues on day 8, we embedded the spheroids in a gel of type I collagen, which represents up to 90% of the cardiac extracellular matrix (ECM) in vivo^14^ (Fig. 1a).

**Extended Data Fig. 1:**
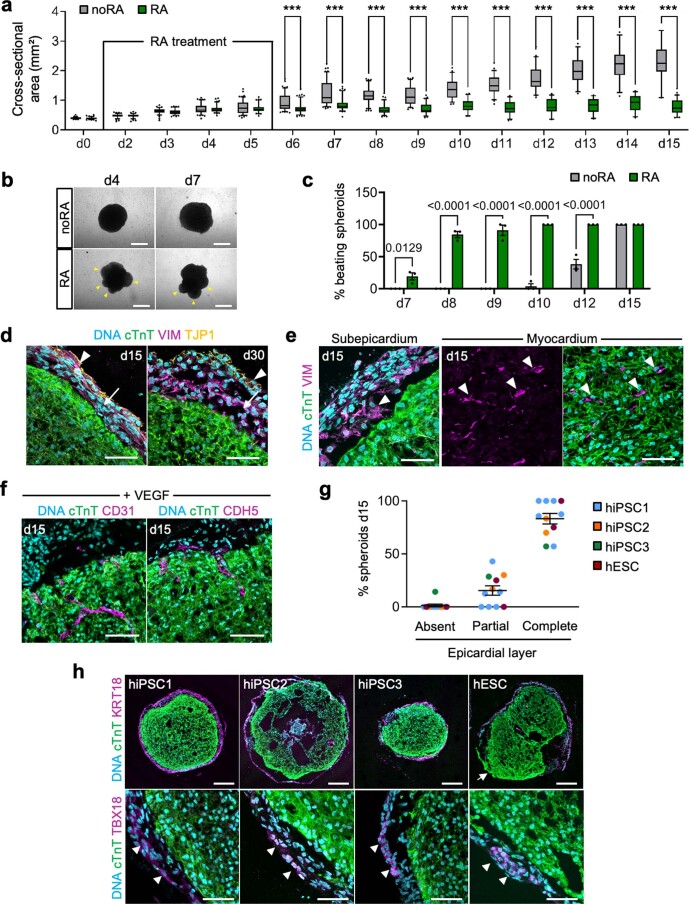
Characterization of 3D cardiac induction with or without retinoic acid. **(a)** Cross-sectional area of spheroids during 3D cardiac induction with retinoic acid (RA) or without (noRA). Box plots indicate the median, 25^th^ and 75^th^ percentile, with whiskers extending to the 5^th^ and 95^th^ percentiles. N per day provided in the Source Data; 3 independent differentiations/group; two-way ANOVA with Sidak’s multiple comparisons test, ***p < 0.0001. **(b)** Representative bright field images of spheroids at days 4 and 7 of differentiation. Yellow arrowheads indicate rounded protrusions typical of RA spheroids. Scale bars = 500 µm. **(c)** Percentage of beating spheroids at the indicated days of differentiation; mean ± SEM. noRA: N = 55 spheroids, RA: N = 96 spheroids; 3 independent differentiations/group. Two-way ANOVA with Sidak’s multiple comparisons test. **(d)** Immunostaining for cTnT, TJP1 and VIM in epicardioids (days 15 and 30). Arrowheads = mesothelial epicardium; arrows = epicardium-derived cells. Scale bars = 50 µm. **(e)** Immunostaining for cTnT and VIM in epicardioids (day 15). Arrowheads indicate examples of cTnT^−^ VIM^+^ mesenchymal cells in the subepicardial space (left) and within the myocardium (right). Scale bars = 50 µm. **(f)** Immunostaining for cTnT and CD31 or CDH5 in VEGF-treated epicardioids (day 15). Scale bars = 50 µm. Images in (d-f) are representative of 3 independent differentiations. **(g,h)** Percentage of spheroids with an absent, partial or complete epicardial layer at day 15 of epicardioid differentiation of different human induced pluripotent (hiPSC) or embryonic stem cell (hESC) lines. The starting cell number was adapted to the cell lines’ proliferation rate to obtain a consistent spheroid size at day 0. Mean ± SEM; hiPSC1: N = 6 independent differentiations, hiPSC2: N = 2, hiPSC3: N = 1, hESC: N = 2; N per differentiation provided in the Source Data. (g) Representative images of cTnT and cytokeratin 18 (KRT18) (top) or TBX18 (bottom) immunostaining of day-15 epicardioids from the different hPSC lines. The arrow indicates an example of a partial epicardial layer, arrowheads show examples of TBX18^+^ epicardial cells. Scale bars top = 200 µm, bottom = 50 µm. (h).

Differences in shape and size quickly appeared between spheroids cultured with and without RA, with the latter growing significantly larger (Fig. 1a and Extended Data Fig. 1a,b). RA-treated spheroids also started spontaneously beating earlier than noRA spheroids (around days 8 and 12, respectively; Extended Data Fig. 1c and Supplementary Videos 1 and 2). Immunofluorescence analysis at day 15 of differentiation revealed that noRA spheroids were mainly composed of loosely organized CMs, marked by the sarcomeric protein cardiac troponin T (cTnT; Fig. 1b). By contrast, RA-treated spheroids consistently formed an inner core of densely packed CMs and a thick envelope containing cells expressing the epicardial markers WT1, TBX18, BNC1, ALDH1A2 and TCF21, leading us to name them epicardioids (Fig. 1b,c). This epicardial compartment, which was maintained over several weeks, consisted of an outer epithelial layer (KRT18 and TJP1) and subjacent vimentin-positive mesenchymal cells, matching the multilayered structure specific to the ventricular epicardium of early human embryos (Fig. 1d and Extended Data Fig. 1d)^15^. The presence of cells expressing the EMT marker TWIST1 in the subepicardial space supported the derivation of EPDCs from mesothelial epicardium (Fig. 1e). Mesenchymal cells interspersed among CMs further suggested migration of EPDCs into the myocardium after EMT (Extended Data Fig. 1e). Having observed poor endothelial contribution, we complemented the maintenance medium with the angiogenic factor vascular endothelial growth factor (VEGF), which was sufficient to obtain vessel-like structures positive for CD31 and vascular endothelial cadherin (CDH5; Extended Data Fig. 1f).

Importantly, the generation of epicardioids was highly reproducible across four hPSC lines, with similar efficiencies in obtaining a complete or partial epicardial layer, overall 83.3% and 15.4%, respectively (Extended Data Fig. 1g,h). Moreover, modulating RA signaling during differentiation indicated a critical window and dosage of RA application for efficient and reproducible epicardium formation (Supplementary Results and Supplementary Fig. 1a–e).

### Analysis of epicardioid composition by single-cell RNA sequencing (scRNA-seq)

**Extended Data Fig. 2:**
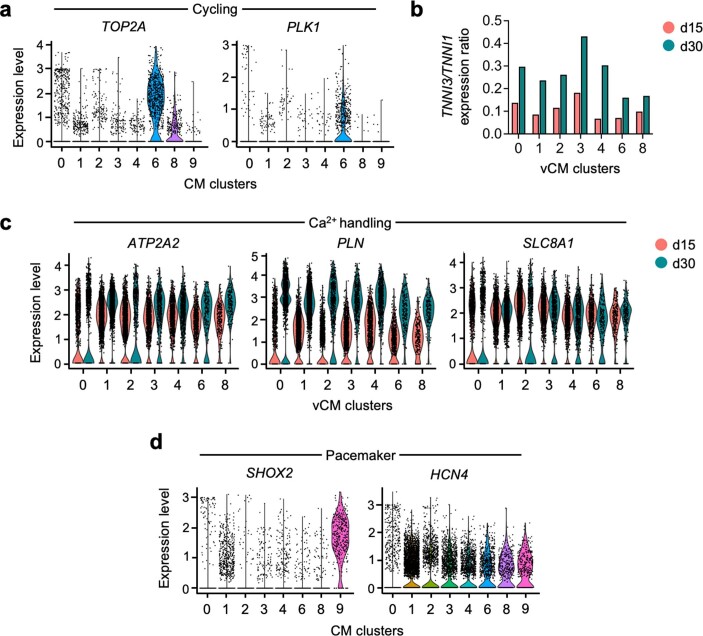
Transcriptional profiles of myocytic clusters in epicardioids at days 15 and 30. **(a)** Violin plots of the expression levels of markers of cycling cells (*TOP2A, PLK1*) in the cardiomyocyte (CM) clusters obtained from the scRNA-seq analysis of epicardioids at days 15 and 30. **(b)** Average expression ratio of the adult to fetal cardiac troponin I isoforms (*TNNI3/TNNI1*) in ventricular CM (vCM) clusters obtained from scRNA-seq analysis of epicardioids at days 15 and 30. **(c)** Violin plots of the expression levels of genes related to CM Ca^2+^ handling (*ATP2A2, PLN, SLC8A1*) in vCM clusters; cells from epicardioids at day 15 and 30 are shown in red and blue, respectively. **(d)** Violin plots of the expression levels of markers of pacemaker CMs (*SHOX2, HCN4*) in the CM clusters obtained from the scRNA-seq analysis of epicardioids at days 15 and 30.

**Extended Data Fig. 3:**
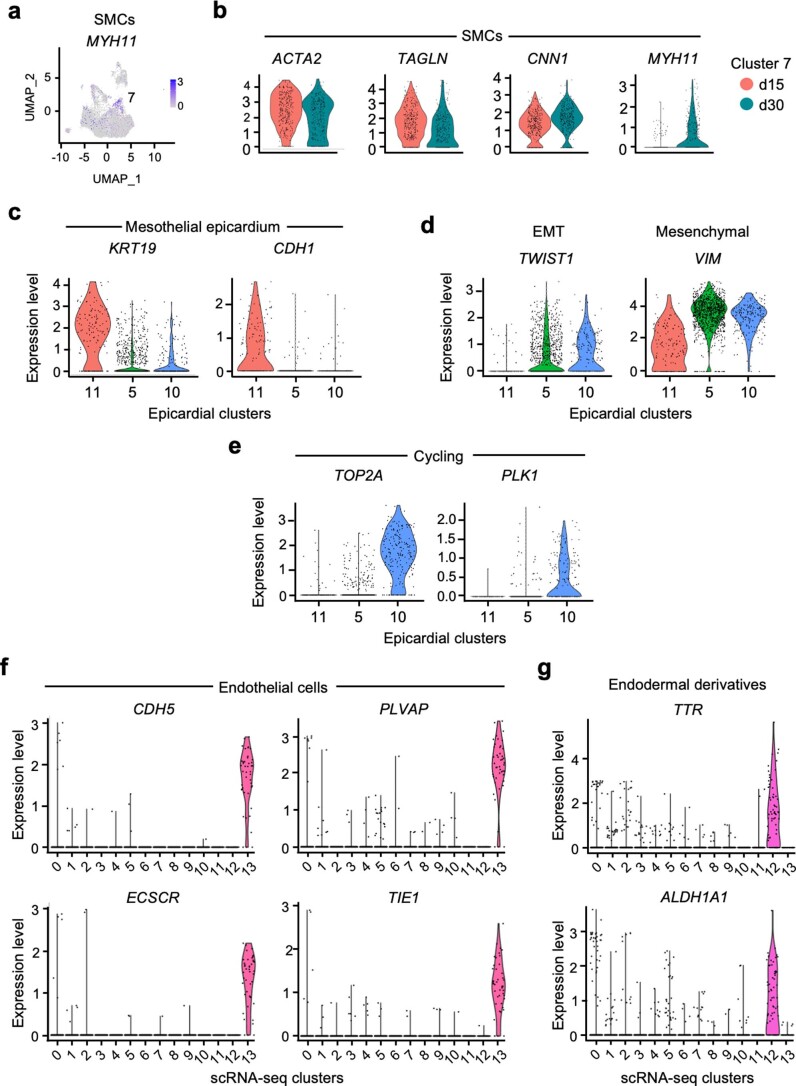
Transcriptional profiles of non-myocytic clusters in epicardioids at days 15 and 30. **(a,b)** Feature plot showing the expression level of the smooth muscle cell (SMC) marker *MYH11* in cells from epicardioids at days 15 and 30. (a) Violin plots showing the expression levels of the SMC markers *ACTA2*, *TAGLN*, *CNN1*, and *MYH11* in the SMC cluster 7, with cells from day 15 and day 30 shown in red and blue, respectively. (b) **(c-e)** Violin plots of the expression levels of markers of mesothelial epicardium (*KRT19, CDH1*) (c), epithelial-to-mesenchymal transition (EMT; *TWIST1*), mesenchymal cells (*VIM*) (d), and cycling cells (*TOP2A, PLK1*) (e) in the epicardial clusters obtained from the scRNA-seq analysis of epicardioids at days 15 and 30. **(f,g)**Violin plots of the expression levels of endothelial cells (*CDH5, PLVAP, ECSCR, TIE1*) (f) and endodermal derivatives (*TTR, ALDH1A1*) (g) in all clusters obtained from scRNA-seq analysis of epicardioids at days 15 and 30.

To further investigate the composition of epicardioids, we performed whole-transcriptome analysis by scRNA-seq at days 15 and 30 (Fig. 1f and Supplementary Table 1). Unsupervised cluster analysis revealed that the most abundant cells were ventricular CMs (vCMs) expressing *MYH7* and *MYL2* (clusters 0, 1, 2, 3 and 4 and proliferative clusters 6 and 8; Fig. 1g and Extended Data Fig. 2a). These cells had a higher ratio of adult-to-fetal cardiac troponin I isoforms (*TNNI3*/*TNNI1*) and increased expression of calcium-handling genes (*ATP2A2, PLN* and *SLC8A1*) at day 30 than at day 15, indicating progressive maturation^16,17^ (Extended Data Fig. 2b,c). Interestingly, there was a small CM cluster (cluster 9) showing coexpression of *SHOX2* and *HCN4*, suggesting pacemaker identity (Extended Data Fig. 2d). Cells expressing transcripts of terminally differentiated SMCs (*ACTA2*, *TAGLN* and *CNN1*) were found in cluster 7, with the mature SMC marker *MYH11* appearing at day 30 (Extended Data Fig. 3a,b). By comparison with recently available sequencing datasets from human fetal epicardium^18–20^, we identified three clusters expressing epicardial markers, which we could broadly define as mesothelial epicardium (cluster 11; *KRT19* and *CDH1*), EMT/epicardium-derived mesenchyme (cluster 5; *TWIST1* and *VIM*) and proliferative cells (cluster 10; *TOP2A* and *PLK1*; Extended Data Fig. 3c–e). The remaining populations in epicardioids were endothelial cells (cluster 13; *CDH5*, *PLVAP*, *ECSCR* and *TIE1*) and endodermal derivatives (cluster 12; *TTR* and *ALDH1A1*; Extended Data Fig. 3f,g).

**Extended Data Fig. 4:**
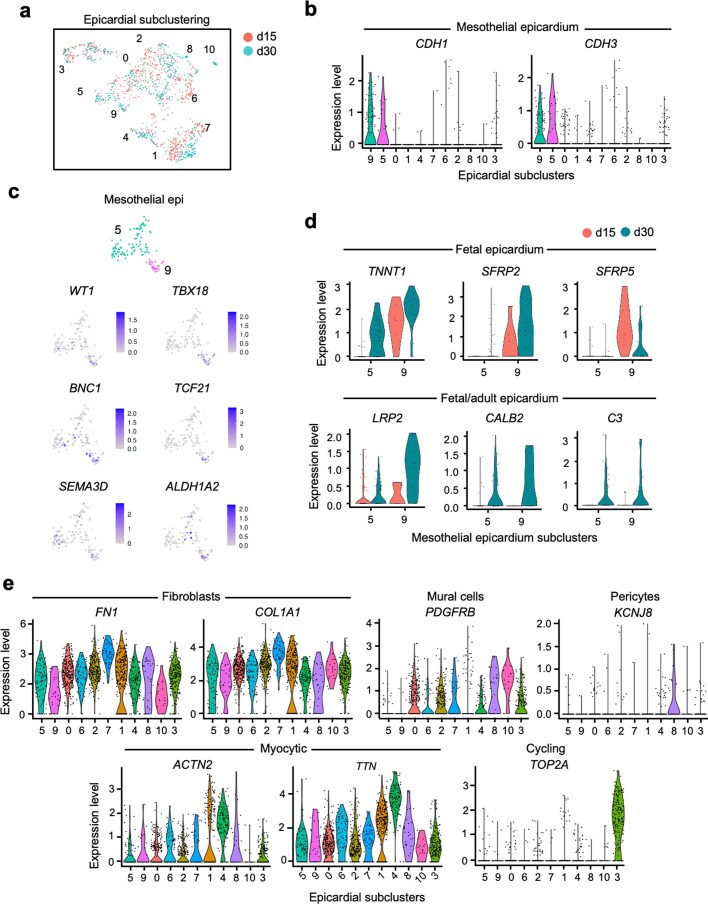
Transcriptional profiles of epicardial populations in epicardioids. **(a)** UMAP plot of the subclustering of the epicardial clusters 11, 5, and 10 obtained from scRNA-seq analysis of epicardioids at day 15 and day 30. Clusters are annotated and cells from day 15 and day 30 are shown in red and blue, respectively. **(b)** Violin plot of the expression levels of the epithelial markers *CDH1* and *CDH3* in the epicardial subclusters. **(c)** Feature plots showing the expression levels of the canonical epicardial markers *WT1*, *TBX18*, *BNC1, TCF21*, *SEMA3D*, and *ALDH1A2* in the mesothelial epicardium subclusters 9 and 5. **(d)** Violin plots showing the expression levels of markers of human epicardium in the mesothelial epicardium subclusters 9 and 5. *TNNT1*, *SFRP2*, and *SFRP5* are expressed in fetal epicardium; *LRP2*, *CALB2*, and *C3* are shared by fetal and adult epicardium. **(e)** Violin plots of the expression levels of markers of fibroblasts (*FN1*, *COL1A1*), mural cells (*PDGFRB*), pericytes (*KCNJ8*), cardiomyocytes (*ACTN2*, *TTN*), and cycling cells (*TOP2A*) in all epicardial subclusters.

We further investigated the heterogeneity of epicardial cells by performing subclustering and inferring cellular dynamics based on the kinetics of gene expression via RNA velocity^21^ (Fig. 1h, Extended Data Fig. 4a and Supplementary Table 2). This revealed two mesothelial populations (subclusters 9 and 5; *KRT19*, *CDH1* and *CDH3*) with heterogeneous expression of the canonical epicardial markers *WT1*, *TBX18*, *BNC1*, *TCF21*, *SEMA3D* and *ALDH1A2*, as described in vivo and in vitro^11,22–25^ (Fig. 1h,i and Extended Data Fig. 4b,c). Subcluster 9 showed stronger upregulation of specific markers of fetal human epicardium (*TNNT1*, *SFRP2* and *SFRP5*), including some shared by fetal and adult epicardium (*LRP2*, *CALB2* and *C3*)^20^, suggesting higher maturation (Extended Data Fig. 4d). We also identified EPDCs in various stages of differentiation based on the expression levels of EMT genes and well-established markers of epicardial derivatives. We could distinguish between uncommitted EPDCs (subclusters 0 and 6), EPDCs differentiating into fibroblasts (subclusters 2 and 7; *TNC*, *FN1* and *COL1A1*) and EPDCs differentiating into mural cells (subclusters 8 and 10; *RGS5* and *PDGFRB*), with subcluster 8 specifically expressing pericyte-related genes (*MCAM* and *KCNJ8*; Fig. 1h,i and Extended Data Fig. 4e). Interestingly, subcluster 1 contained EPDCs expressing both fibroblast (*FN1* and *COL1A1*) and CM markers (*TNNT2*, *TTN* and *ACTN2*), and the latter genes were further upregulated in subcluster 4, suggesting myocytic differentiation (Fig. 1h,i and Extended Data Fig. 4e). Epicardial cells with high expression of cardiac sarcomeric genes have not yet been reported in hPSC-based two-dimensional (2D) epicardial differentiation models^9,11,26^. Indeed, the transcriptional signatures of 2D epicardial cells described by Gambardella et al.^11^ specifically marked mesothelial cells of subclusters 5 and 9 (*BNC1*^high^ signature) and EPDCs of subclusters 0 and 2 (*TCF21*^high^ signature; Supplementary Fig. 2a,b). Moreover, markers of fetal human epicardium found in subclusters 5 and 9 were low or absent in 2D cells (Supplementary Fig. 2c), suggesting further epicardial development in the three-dimensional (3D) environment of epicardioids.

**Extended Data Fig. 5:**
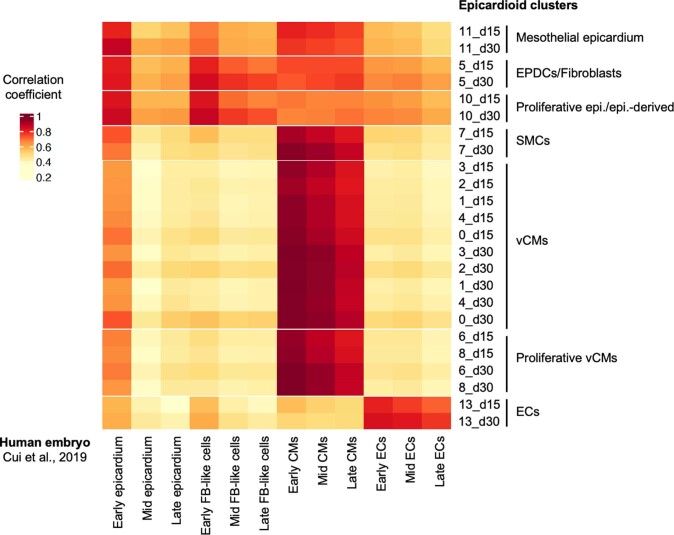
Correlation of the developmental stage of epicardioids with human fetal development. Correlation analysis between the indicated epicardioid clusters at days 15 or 30 and corresponding cell populations from human embryos analyzed by scRNA-seq by Cui et al., 2019. The early, mid, and late stages are defined as 5-7 weeks, 9-17 weeks, and 20-25 weeks of embryonic development, respectively. Correlation coefficient values are represented by the indicated color legend. EPDCs: epicardium-derived cells, Epi.: epicardium, ECs: endothelial cells, FB-like: fibroblast-like.

Having observed signs of progressive maturation in different cell types, we sought to benchmark the developmental stage of epicardioids against human cardiogenesis. Transcriptomic correlation analysis using a human fetal dataset^18^ revealed that cells in epicardioids at day 15 correlated with their in vivo counterparts from early stages of development (5–7 weeks; Extended Data Fig. 5). By day 30, there was increased correlation with mid-stage and late-stage fetal development (9–17 and 20–25 weeks, respectively; Extended Data Fig. 5).

### Epicardioids recapitulate human ventricular patterning

**Fig. 2:**
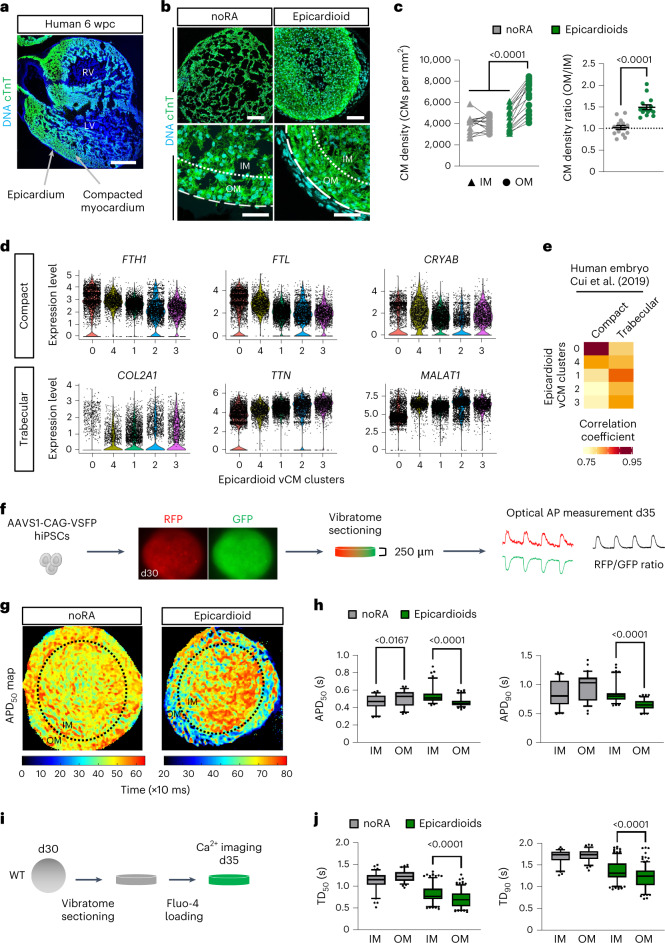
Epicardioids display morphological, molecular and functional self-patterning of the myocardium. **a**,**b**, Immunostaining for cTnT in human ventricular tissue (6 weeks postconception (wpc); **a**) and in noRA spheroids and epicardioids (day 15; **b**). Images are representative of three independent samples and six differentiations, respectively; RV, right ventricle; LV, left ventricle. In **b**, dashed lines indicate the outer edge of the myocardium, and dotted lines indicate separation between the OM (50 µm wide) and IM; scale bars in **a**, 500 µm; scale bars in **b**, 100 µm (top) and 50 µm (bottom). **c**, Left, CM density in the OM and IM of noRA spheroids and epicardioids (day 15). For all data points, lines connect values from the same sample; *n* = 15 spheroids and 3 independent differentiations per group. Data were analyzed by two-way ANOVA with a Tukey’s multiple comparisons test. Right, ratio of CM density OM to IM. Data are shown as mean ± s.e.m.; *n* = 15 spheroids and 3 independent differentiations per group. Data were analyzed by unpaired two-tailed *t*-test. **d**, Expression levels of compact and trabecular markers in scRNA-seq vCM clusters from epicardioids (days 15 and 30). **e**, Correlation analysis between vCM clusters and compact or trabecular CMs from human embryos^18^ (coefficients are in pseudocolor). **f**, Three-dimensional differentiation of hiPSCs expressing a FRET-based voltage indicator (AAVS1-CAG-VSFP) for optical action potential (AP) measurement in 250-µm-thick slices at day 35. **g**, Representative map of APD_50_ in a noRA spheroid and epicardioid (day 35). **h**, APD_50_ (left) and APD_90_ (right) in the OM and IM of noRA spheroids and epicardioids under 0.5-Hz pacing (day 35); *n* = 60 action potentials per layer and 3 noRA spheroids; *n* = 100 action potentials per layer and 6 epicardioids. Three independent differentiations per group were performed. Data were analyzed by Kruskal–Wallis test with a Dunn’s multiple comparisons test. **i**, Calcium transient imaging in 250-µm-thick slices cut at day 30 and loaded with Fluo-4 at day 35. **j**, Transient duration at 50% (TD_50_; left) and transient duration at 90% (TD_90_; right) in the OM and IM of noRA spheroids and epicardioids under 0.5-Hz pacing (day 35); *n* = 75 transients per layer and 4 noRA spheroids; *n* = 200 transients per layer and 9 epicardioids. Three independent differentiations per group were performed. Data were analyzed by Kruskal–Wallis test with a Dunn’s multiple comparisons test. Box plots in **h** and **j** indicate the median and 25th and 75th percentiles, with whiskers extending to the 5th and 95th percentiles.

A major step of ventricular morphogenesis is the formation of a subepicardial compact myocardium layer that is molecularly and functionally distinct from the trabecular myocardium facing the ventricular lumen. This organization is already clearly visible at 6 weeks after conception in human embryos (Fig. 2a). In epicardioids, we noted the presence of an approximately 50-µm-wide zone of increased CM density underneath the epicardium from day 15, with a mean density ratio of 1.49 between this outer layer and the inner myocardium (IM; Fig. 2b,c). This was not observed in spheroids differentiated without RA, which had a mean density ratio of 1.02 (Fig. 2b,c). In our scRNA-seq dataset, opposing gradients in the expression levels of genes enriched in human compact (*FTH1*, *FTL* and *CRYAB*) and trabecular (*COL2A1*, *TTN* and *MALAT1*) myocardium also suggested a molecular patterning of vCMs in epicardioids, which was supported by correlation analysis with compact and trabecular cells from human embryos^18^ (Fig. 2d,e).

This prompted us to evaluate if epicardioids harbor regional differences in CM function. In vivo, CMs closest to the epicardium generate shorter action potentials than those in the middle of the ventricular wall; this is an evolutionarily conserved feature known as the transmural voltage gradient, which increases the efficiency of ventricular contraction^27^. We assessed action potential dynamics in the myocardial compartment of epicardioids generated from human induced PSCs (hiPSCs) constitutively expressing a FRET-based voltage sensor knocked into the AAVS1 safe harbor locus^28,29^ (AAVS1-CAG-voltage-sensitive fluorescent protein (AAVS1-CAG-VSFP); Fig. 2f). Optical measurements of AP duration in epicardioid slices revealed significantly shorter durations to 50% and 90% repolarization in CMs of the subepicardial layer than in CMs of the IM at day 35 (Fig. 2g,h). As excitation–contraction coupling is dependent on the intracellular dynamics of calcium, we additionally performed calcium imaging with the fluorescent indicator Fluo-4 (Fig. 2i). This showed a corresponding pattern of shorter durations to 50% and 90% peak decay in the subepicardial layer than in the inner layer (Fig. 2j). Neither of these functional gradients were observed in age-matched noRA spheroids, confirming that they are not intrinsic properties of cardiac spheres (Fig. 2g,h,j).

### Investigating the functional cross-talk between cardiac cells

**Fig. 3:**
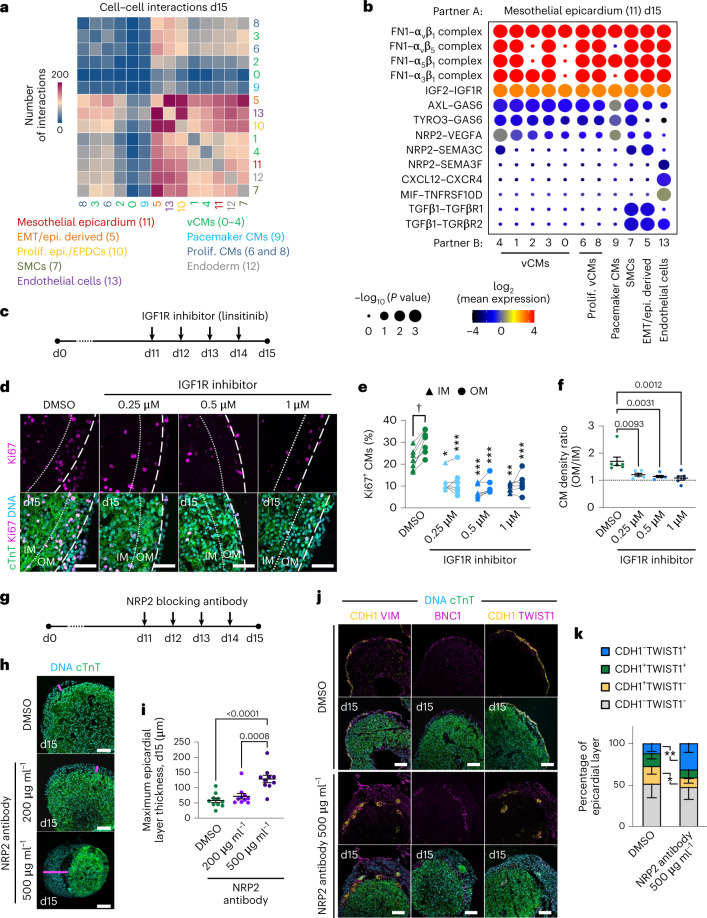
Epicardioids shed light on the cellular cross-talk regulating human myocardial and epicardial development. **a**, Number of cell–cell interactions inferred between all scRNA-seq clusters in epicardioids (day 15). **b**, Selected interactions between mesothelial epicardium and the indicated clusters in day 15 epicardioids. Circle size indicates the two-tailed permutation-based *P* values, and color indicates the mean expression level of the interacting molecules. **c**,**d**, Epicardioids were treated with DMSO or the IGF1R inhibitor linsitinib from days 11 to 15 (**c**). Representative images of immunostaining for cTnT and Ki67 in treated and control epicardioids (day 15) were acquired (**d**); scale bars, 50 µm. **e**, Percentage of Ki67^+^ CMs in the OM and IM of treated and control epicardioids (day 15). Lines connect the values for OM and IM within the same sample. Data were analyzed by two-way ANOVA with a Tukey’s multiple comparisons test. Asterisks indicate *P* values from comparing the OM or IM of treated samples with the corresponding layer of controls; ^†^*P* = 0.02; *P = 0.004; **P = 0.0002; ***P < 0.0001. **f**, Ratio of CM density OM to IM in treated and control epicardioids (day 15). Data are shown as mean ± s.e.m. and were analyzed by one-way ANOVA with a Tukey’s multiple comparisons test; DMSO, *n* = 7 epicardioids; linsitinib, *n* = 6 epicardioids per concentration. Three independent differentiations per group were performed. **g**–**i**, Epicardioids were treated with DMSO or an NRP2 blocking antibody from days 11 to 15 (**g**). Representative images of cTnT immunostaining in treated and control epicardioids (day 15) are shown (**h**). Pink lines indicate maximum epicardium thickness; scale bars, 100 µm. Maximum epicardium thickness in epicardioids of each condition (day 15) were calculated (**i**). Data are presented as mean ± s.e.m. and were analyzed by one-way ANOVA with a Tukey’s multiple comparisons test; *n* = 10 epicardioids and 3 independent differentiations per group. **j**,**k**, Representative images of immunostaining for cTnT, CDH1 and vimentin, cTnT and BNC1 or cTnT, CDH1 and TWIST1 in day 15 epicardioids treated with DMSO or 500 µg ml^–1^ NRP2 blocking antibody were acquired (**j**); scale bars, 100 µm. The percentage of cells expressing CDH1 and/or TWIST1 in the epicardial layer of treated and control epicardioids (day 15) was calculated (**k**). Data are presented as means ± s.d. and were analyzed by unpaired two-tailed *t*-test; *P = 0.03; **P = 0.002; *n* = 9 epicardioids and 3 independent differentiations per group.

**Extended Data Fig. 6:**
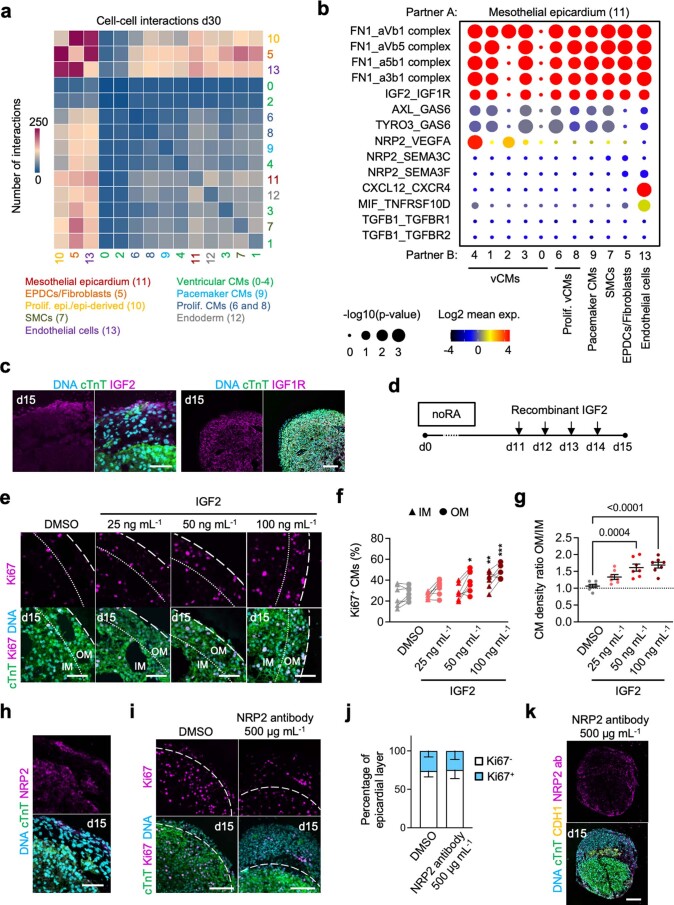
Investigating cell-cell communications in epicardioids. **(a)** Number of cell-cell interactions inferred between all scRNA-seq clusters in epicardioids (day 30). **(b)** Selected interactions between mesothelial epicardium and the indicated clusters (day 30). Circle size = two-tailed permutation-based p-values, color = mean of the expression level of the interacting molecules. **(c)** Immunostaining for cTnT and IGF2 (left) or IGF1R (right) in day-15 epicardioids (representative of 2 independent differentiations). Scale bar left = 50 µm, right = 100 µm. **(d)** Spheroids differentiated without retinoic acid (noRA) were treated with DMSO or IGF2 from days 11 to 15. **(e)** Representative images of immunostaining for cTnT and Ki67 in treated and control noRA spheroids (day 15). Hatched lines = outer edge of the myocardium; dotted lines = separation between the OM and IM. Scale bars = 50 µm. **(f)** Percentage of Ki67^+^ CMs in the OM and IM of treated and control noRA spheroids (day 15). All data points; lines connect the values for OM and IM within the same sample. Two-way ANOVA with Tukey’s multiple comparisons test; *p = 0.01; **p = 0.001; ***p < 0.0001; asterisks indicate the p-values obtained from comparing the OM or IM of treated samples with the corresponding layer of controls. N = 7 spheroids, 3 independent differentiations/group. **(g)** Ratio of CM density (CMs/mm^2^) OM/IM in treated and control noRA spheroids (day 15). Mean ± SEM; one-way ANOVA with Tukey’s multiple comparisons test. N = 7 spheroids, 3 independent differentiations/group. **(h)** Immunostaining for cTnT and NRP2 in day-15 epicardioids (representative of 2 independent differentiations). Scale bar = 50 µm. **(i)** Representative images of immunostaining for cTnT and Ki67 in day-15 epicardioids treated with DMSO or 500 µg/mL NRP2 blocking antibody. Scale bars = 100 µm. **(j)** Percentage of Ki67^+^ and Ki67^−^ epicardial cells in treated or control epicardioids (d15). Mean ± SD. N = 9 epicardioids, 3 independent differentiations/group. **(k)** NRP2 blocking antibody targeted with a secondary antibody alongside immunostaining for cTnT and CDH1 in day-15 epicardioids treated with 500 µg/mL NRP2 blocking antibody (representative of 2 independent differentiations). Scale bar = 100 µm.

Studies in animals have shown that signals emanating from the epicardium regulate myocardium development and vice versa, but corresponding data in humans are scarce. Inferring cell–cell communications in our scRNA-seq dataset using CellphoneDB^30^ suggested ample interaction between the epicardium and other cell types, with a slight decrease from day 15 to day 30 (Fig. 3a and Extended Data Fig. 6a). We detected epicardial signals known to stimulate CM proliferation in the mouse, such as the secretion of IGF2 and fibronectin binding to integrin-β1 as well as the CXCL12–CXCR4 axis promoting coronary angiogenesis in zebrafish^3,31–33^ (Fig. 3b and Extended Data Fig. 6b). We also noted ligand–receptor interactions recently described between epicardial cells and CMs (NRP2–VEGFA), endothelial cells (MIF–TNFRSF10D) and fibroblasts (NRP2–SEMA3C) in human embryos^20^ (Fig. 3b and Extended Data Fig. 6b). At day 15, we found epicardial TGFβ1 binding to TGFβR1/TGFβR2 in EPDCs/fibroblasts and SMCs, a signaling pathway that is known to be involved in epicardial EMT and EPDC differentiation into SMCs^34,35^ (Fig. 3b).

We took a particular interest in the interaction between epicardial IGF2 and myocytic IGF1R, which was identified as the primary driver of myocardial compaction in rodents but has not yet been studied in a human system^3,33,36,37^. After confirming the protein expression of IGF2 and IGF1R in epicardial cells and CMs, respectively (Extended Data Fig. 6c), we treated epicardioids with increasing concentrations of the small-molecule IGF1R inhibitor linsitinib from days 11 to 15 (Fig. 3c). In DMSO-treated controls, immunostaining for the cell cycle activity marker Ki67 (ref. ^38^) at day 15 revealed significantly higher CM proliferation in the compact outer myocardium (OM) than in the IM, in line with higher mitotic activity in the compact layer during development^39^ (Fig. 3d,e). Linsitinib treatment dramatically reduced the percentage of proliferating CMs in both layers at every concentration applied (Fig. 3d,e). This was associated with a decrease in the CM density ratio between the OM and IM, indicating a failure of subepicardial compaction (Fig. 3f). The opposite effects were observed when treating noRA spheroids (which lack the epicardial layer) with recombinant human IGF2 (Extended Data Fig. 6d). IGF2 treatment caused a dose-dependent increase in proliferation in the OM, which was associated with a significantly higher CM density ratio between the OM and IM (Extended Data Fig. 6e–g). This suggested that IGF2 was sufficient to induce myocardial compaction in the absence of an epicardium.

We next focused on epicardial NRP2, which is predicted to interact with ligands from different cell types, including CMs, fibroblasts and endothelial cells, in epicardioids and human embryos^20^. NRP2 is widely expressed in the heart and was first implicated in embryonic neuronal guidance and angiogenesis^40^. NRP2 was also found to be upregulated in the epicardium following cardiac injury in adult zebrafish, but its role in the developing epicardium is not clear^41^. In epicardioids, NRP2 protein was detected in the mesothelial epicardium and in CMs but not in cells of the subepicardial space (Extended Data Fig. 6h). To perturb NRP2 activity, we treated epicardioids with a blocking antibody to NRP2 from days 11 to 15 (Fig. 3g). The highest concentration led to a significant thickening of the epicardial layer by day 15 compared to DMSO-treated controls (Fig. 3h,i). In these samples, the CDH1^+^BNC1^+^ mesothelial layer was disrupted, and it lay in close contact with the myocardium, while large numbers of mesenchymal cells were oriented outward (Fig. 3j). This reflected an increased percentage of CDH1^–^TWIST1^+^ cells and a decrease of CDH1^+^TWIST1^–^ cells but no change in proliferation compared to controls (Fig. 3k and Extended Data Fig. 6i,j), suggesting that the thickening of the epicardial layer was caused by excessive epicardial EMT (leading to partial loss of the mesothelial layer) rather than hyperproliferation. The inverted orientation of cells additionally pointed to a defect in cell migration, a process that is regulated by NRP2 signaling in tumor cells of several cancer types^42^. Of note, immunodetection of the NRP2 blocking antibody after treatment indicated its predominant localization in the epicardial layer, suggesting that it did not reach the myocardium (Extended Data Fig. 6k).

### Epicardioids arise from first heart field (FHF) and juxtacardiac field (JCF) progenitors

Animal models have shown that the epicardium is formed by cells of the proepicardium, a transient structure located at the venous pole of the looping-stage heart, but the ontogeny of proepicardial precursors is still unclear, and even less is known about their human counterparts. Building on earlier work of Lescroart et al., who first uncovered a developmental relationship between the myocardium and the epicardium at gastrulation^43^, two studies have recently identified a common progenitor pool of the two lineages located at the rostral border of the cardiac crescent in the mouse^44,45^. Tyser et al. coined the term JCF and showed that JCF cells characterized by *Mab21l2* expression give rise to both epicardial cells and CMs^44^. In parallel, Zhang et al. identified an equivalent *Hand1*^+^ progenitor population and confirmed by clonal lineage tracing that at least some of these cells are bipotent for epicardium and myocardium^45^. Both groups concluded that the JCF likely represents a previously unrecognized subset of the FHF, which mainly produces CMs of the left ventricle in the mouse, but it is not known if this population exists in humans.

**Fig. 4:**
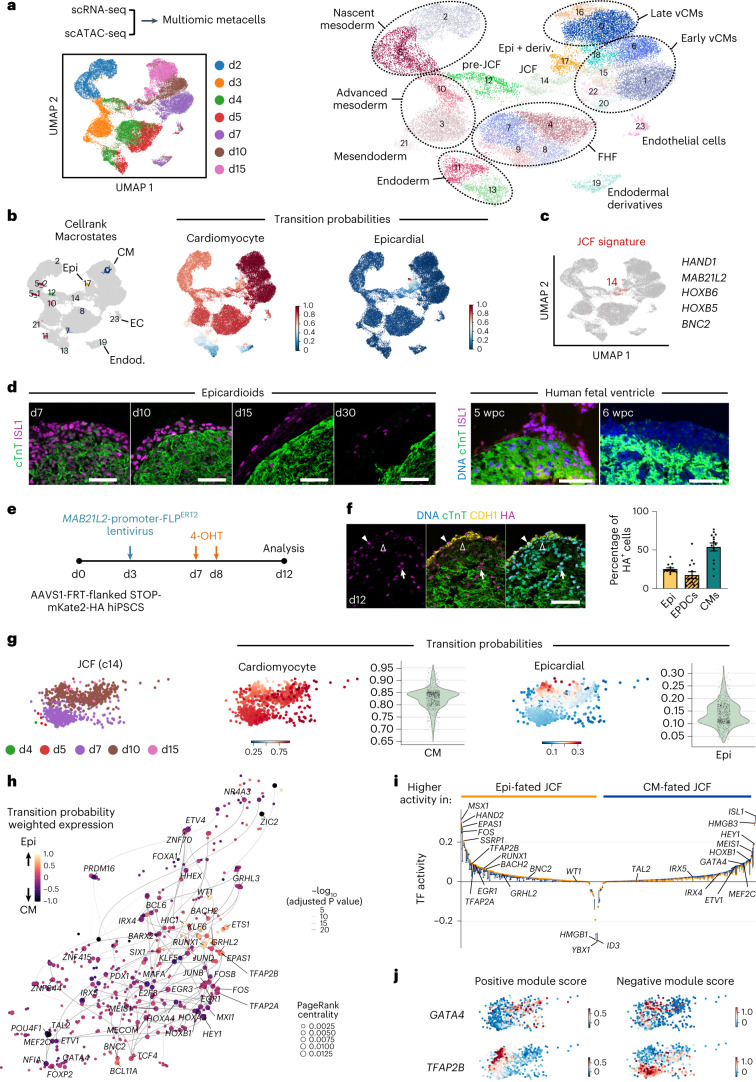
Epicardioids are formed by early segregation of FHF and JCF progenitors. **a**, UMAP plots of multiomic metacells constructed from paired scRNA-seq and scATAC-seq analysis of epicardioids at days 2, 3, 4, 5, 7, 10 and 15, labeled by day (left) and cluster identity (right). **b**, Left, UMAP plot showing the macrostates inferred using CellRank (day 15 states annotated); Epi, epicardial. Right, UMAP plots showing the transition probabilities for the CM and epicardial states. **c**, UMAP plot showing metacells coexpressing JCF markers in red. **d**, Immunostaining for cTnT and ISL1 in epicardioids at days 7, 10, 15, and 30 (left) and in ventricular tissue from human embryos at 5 and 6 weeks after conception (right). Images are representative of three independent differentiations or samples; scale bars, 50 µm. **e**, To trace JCF cells, epicardioids differentiated from AAVS1-FRT-flanked STOP-mKate2-HA reporter hiPSCs were transduced with a lentiviral vector encoding inducible FLP (FLP^ERT2^) under the control of the *MAB21L2* promoter at day 3 and were treated with 4-OHT at days 7 and 8 before analysis at day 12. **f**, Left, representative images of immunostaining for cTnT, CDH1 and the HA tag in infected organoids (day 12). Filled arrowheads indicate HA-tag^+^ mesothelial epicardial cells (epi.), empty arrowheads indicate HA-tag^+^ EPDCs, and arrows indicate HA-tag^+^ CMs; scale bar, 50 µm. Right, percentage of epicardial cells, EPDCs and CMs among HA-tag^+^ cells. Data are shown as mean ± s.e.m.; *n* = 14 epicardioids and 3 independent differentiations. **g**, Left, UMAP plots showing the JCF cluster 14 colored by day. Right, transition probabilities for CM and epicardial states in the JCF cluster. **h**, UMAP embedding of the inferred TF network in the JCF cluster. Node size indicates PageRank centrality, and color indicates transition probability weighted expression. **i**, TFs with varying activity between JCF cells with high transition probability for the CM (blue) and epicardial fate (orange). TF activity is indicated by a colored dot for each fate; positive, mainly activating; negative, mainly repressing. **j**, Module scores for *GATA4* and *TFAP2B* in the JCF cluster.

**Extended Data Fig. 7:**
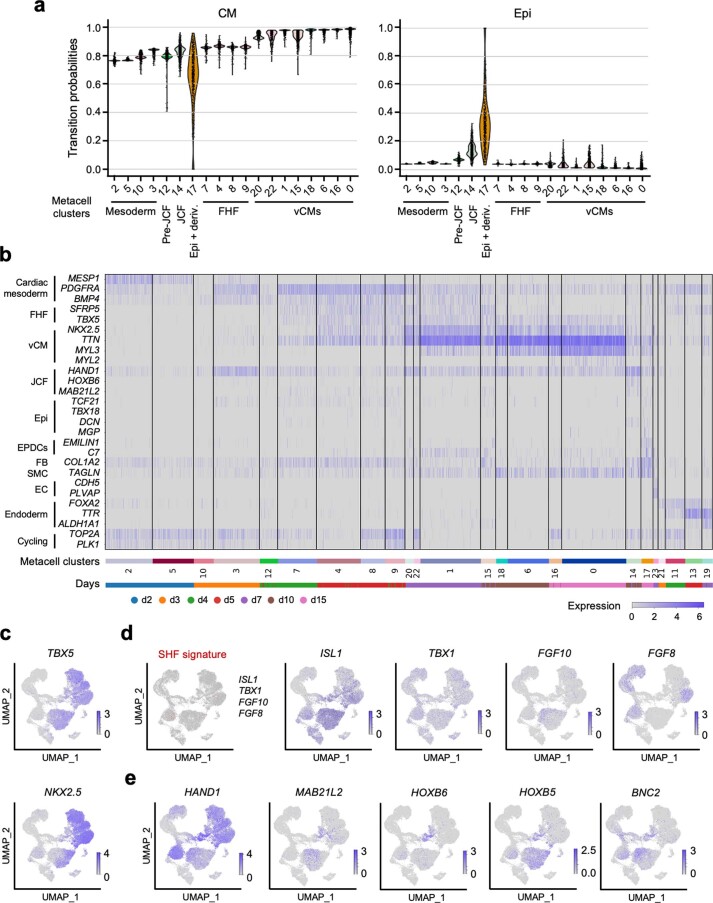
Transcriptional dynamics during epicardioid development. **(a)** Violin plots of the transition probabilities for the myocytic (CM, left) and epicardial (epi, right) macrostates in the indicated metacell clusters obtained from paired scRNA-seq and scATAC-seq analysis of epicardioids at days 2, 3, 4, 5, 7, 10, and 15. **(b)** Heatmap showing the expression of markers of the indicated cell types in all metacell clusters; the legend below indicates from which day each cell originates. FHF: first heart field; vCM: ventricular cardiomyocytes; JCF: juxta-cardiac field; Epi: epicardium; EPDCs: epicardium-derived cells; FB: fibroblasts; SMC: smooth muscle cells; EC: endothelial cells. **(c)** Feature plot showing the expression levels of the FHF markers *TBX5* and *NKX2.5*. **(d)** UMAP plot showing cells co-expressing the anterior second heart field (SHF) markers *ISL1*, *TBX1, FGF8*, and *FGF10* in red (left) and individual feature plot for each gene (right). **(e)** Feature plots of the JCF markers *HAND1*, *MAB21L2*, *HOXB6*, *HOXB5*, and *BNC2*.

To dissect the developmental processes taking place in epicardioids, we performed scRNA-seq in parallel with chromatin accessibility profiling via single-cell assay for transposase-accessible chromatin with sequencing (scATAC-seq) at days 2, 3, 4, 5, 7, 10 and 15 of differentiation. Using the computational method graph-linked unified embedding (GLUE)^46^, we integrated the two modalities by constructing metacells containing both transcriptome and chromatin accessibility information, defining a total of 24 clusters from 35,499 metacells (Fig. 4a and Supplementary Table 3). To follow cell trajectories over time, we used CellRank^47^ to infer the terminal macrostates (myocytic, epicardial, endothelial and endodermal) and the probability of each cell to transition toward these states based on both pseudotime and RNA velocity information (Fig. 4b and Extended Data Fig. 7a). Along the myocytic trajectory, we detected the early induction of cardiac mesoderm (*MESP1*, *PDGFRA* and *BMP4*; days 2 and 3) followed by the emergence of cells expressing markers of FHF progenitors (*TBX5*, *NKX2.5* and *SFRP5*; days 4 and 5; Fig. 4b and Extended Data Fig. 7a–c). Of note, cells coexpressing markers of anterior second heart field (SHF) precursors (*ISL1*, *TBX1*, *FGF8* and *FGF10*), which generate the right ventricle and the outflow tract, were virtually absent (Extended Data Fig. 7d). Differentiated ventricular CMs were detected from day 7 and showed upregulation of the mature ventricular marker *MYL2* by day 15 (Extended Data Fig. 7b). Epicardial cells and their derivatives first emerged on day 10 and expanded on day 15 (cluster 17; Extended Data Fig. 7b). Strikingly, the epicardial trajectory included cells that closely matched the transcriptional signature of the JCF (*HAND1*, *MAB21L2*, *HOXB6*, *HOXB5* and *BNC2*), mainly present at days 7 and 10 (cluster 14; Fig. 4b,c and Extended Data Fig. 7a,b,e). These cells were preceded by putative ‘pre-JCF’ precursors (cluster 12), which appeared on days 4 and 5 at the same time as classical FHF cells (Fig. 4a and Extended Data Fig. 7a). Of note, pre-JCF cells expressed the multipotent cardiovascular progenitor marker *ISL1* but not the myocytic marker *NKX2.5* (ref. ^48^; Supplementary Fig. 3a). This allowed us to follow their physical segregation from ISL1^+^NKX2.5^+^ FHF progenitors, which was clearly visible by day 5, with pre-JCF cells already concentrated at the outer layer (Supplementary Fig. 3b). *ISL1* was still highly expressed in the JCF, followed by a downregulation in the epicardial cluster (Supplementary Fig. 3c). This reflected the maintenance of ISL1 in the mesothelial epicardium but in very few EPDCs at day 15 (Fig. 4d). As ISL1 expression was not previously reported in the epicardium (human or otherwise), we performed ISL1 immunostaining in human fetal heart tissue to verify this finding. This revealed that epicardial cells were indeed positive for ISL1 at 5 weeks after conception, but expression was lost by 6 weeks (Fig. 4d). We also observed decreased ISL1 expression in epicardial cells of 30-day-old epicardioids, suggesting equivalent expression dynamics (Fig. 4d).

### The human JCF gives rise to both epicardium and myocardium

**Extended Data Fig. 8:**
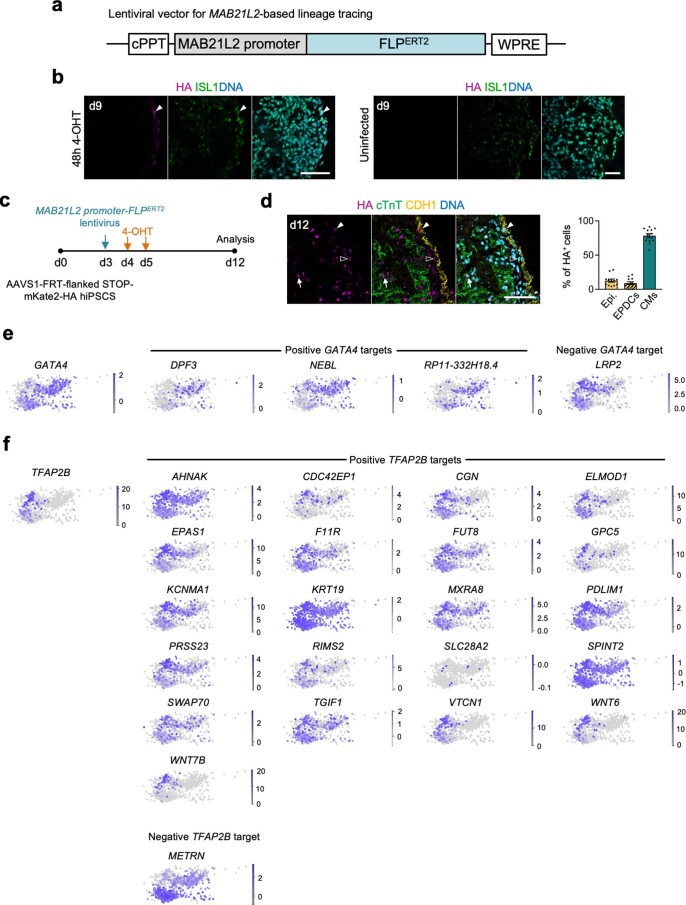
Analysis of JCF potential by lineage tracing and multi-omic analysis. **(a)** Lentiviral vector used for lineage tracing of JCF progenitors in epicardioids derived from AAVS1-CAG-FRT-flanked STOP-mKate2-HA reporter hiPSCs, consisting of the sequence encoding Tamoxifen-inducible flippase (FLP^ERT2^) driven by the *MAB21L2* promoter. cPPT: central polypurine tract; WPRE: woodchuck hepatitis virus posttranscriptional regulatory element. **(b)** (Left) Immunostaining for the HA-tag and ISL1 in epicardioids at day 9, after infection with the MAB21L2-FLP^ERT2^ lentivirus at day 3 and application of hydroxtamoxifen (4-OHT) days 7-8. The arrowhead shows an exemplary HA-tag^+^ ISL1^+^ JCF cell at the outer layer. (Right) Corresponding uninfected negative control. Images are representative of 3 independent differentiations. **(c,d)** Experimental protocol used for lineage tracing of early *MAB21L2*^*+*^ progenitors. Epicardioids differentiated from AAVS1-FRT-flanked STOP-mKate2-HA reporter hiPSCs were transduced with a lentiviral vector encoding inducible flippase (FLP^ERT2^) under the control of the *MAB21L2* promoter at day 3 and treated with 4-OHT at days 4 and 5 before analysis at day 12. (c) (Left) Representative images of immunostaining for cTnT, E-cadherin (CDH1) and the HA-tag in infected organoids at day 12. Filled arrowhead: exemplary HA-tag^+^ mesothelial epicardial cell (epi), empty arrowhead: exemplary HA-tag^+^ epicardium-derived cell (EPDC), arrow: exemplary HA-tag^+^ cardiomyocyte (CM). Scale bar = 50 µm. (Right) Percentage of epi, EPDCs and CMs among HA-tag^+^ cells. Mean ± SEM; N = 12 epicardioids, 3 independent differentiations. (d) **(e,f)** UMAP plots showing the expression of the transcription factor (TF) *GATA4* and its downstream targets in the JCF metacell cluster 14 (positively regulated: *DPF3*, *NEBL*, *RP11-332H18.4*; repressed: *LRP2*). (e) UMAP plots showing the expression of the TF *TFAP2B* and its downstream targets in the JCF metacell cluster 14 (positively regulated: *AHNAK*, *CDC42EP1*, *CGN*, *ELMOD1*, *EPAS1*, *F11R*, *FUT8*, *GPC5*, *KCNMA1*, *KRT19*, *MXRA8*, *PDLIM1*, *PRSS23*, *RIMS2*, *SLC28A2*, *SPINT2*, *SWAP70*, *TGIF1*, *VTCN1*, *WNT6*, *WNT7B*; repressed: *METRN*). (f).

To functionally validate the fate potential of different progenitor populations in epicardioids, we generated an hiPSC reporter line in which a flippase (FLP) recognition target (FRT)-flanked neomycin cassette blocking transcription of a pCAG-driven fluorescent reporter (mKate2) fused to a hemagglutinin (HA) tag was knocked into the AAVS1 safe harbor locus^49^ (Supplementary Results and Supplementary Fig. 4a–f). For JCF lineage tracing, epicardioids from this reporter line were transduced with a lentiviral vector encoding an inducible FLP (FLP^ERT2^) under the control of the *MAB21L2* promoter at day 3, and 4-hydroxytamoxifen (4-OHT) was applied at the beginning of the JCF stage (days 7–8; Fig. 4e and Extended Data Fig. 8a). In the absence of a reliable antibody to MAB21L2, we performed co-staining of the HA tag and ISL1 after 48 h to confirm successful labeling of ISL1^+^ JCF cells located at the outer layer (Extended Data Fig. 8b). On day 12, immunofluorescence analysis revealed HA-tag^+^ CMs and epicardial cells (both mesothelial and EPDCs), confirming the dual fate potential of JCF progenitors as seen in the mouse (Fig. 4f). Having also observed *MAB21L2*^*+*^ cells in metacell clusters categorized as FHF progenitors (mainly cluster 9), we alternatively applied 4-OHT at the corresponding stage (days 4–5; Extended Data Fig. 8c). In this case, 78.4% of HA-tag^+^ cells at day 12 were CMs compared to 54.7% when applying 4-OHT at the JCF stage (Fig. 4f and Extended Data Fig. 8d). Considering the close relationship between the JCF and the FHF in the mouse, it is unclear whether (some of) these cells descended from classical FHF progenitors expressing *MAB21L2* or if there exists an early JCF population with higher commitment to the myocytic lineage. Moreover, a closer look at fate trajectories in our metacell dataset revealed heterogeneity in the JCF cluster, with the majority of cells having a high probability for myocytic differentiation but only a subset appearing to be bipotent for the myocytic and epicardial lineages (Fig. 4g). We inferred the gene regulatory networks (GRNs) associated with each fate using Pando^50^, a recently established algorithm leveraging both transcriptome and chromatin accessibility data (Fig. 4h and Supplementary Tables 4 and 5). We identified well-known transcription factors (TFs) involved in CM differentiation (for example, *GATA4*, *ISL1* and *MEIS1*) and uncovered putative drivers of epicardial differentiation of JCF cells (for example, *TFAP2B*, *HAND2* and *FOS*; Fig. 4h,i). The expression patterns of positively and negatively regulated downstream targets of TFs specific of each fate (for example, *GATA4* and *TFAP2B*) clearly indicated distinct regulatory programs reflecting the dual potential of JCF cells (Fig. 4j and Extended Data Fig. 8e,f).

### Exploring the lineage potential of the human epicardium

Beyond their embryonic origin, there are still many open questions concerning the molecular and functional heterogeneity of epicardial cells, which have important implications for epicardial reactivation as a potential therapeutic target^51,52^. Specifically, it is still unclear if mammalian epicardial cells can give rise to CMs and whether the lineage fate of EPDCs is predetermined at the (mesothelial) epicardial stage or if specification occurs after EMT^22,23,25^.

**Fig. 5:**
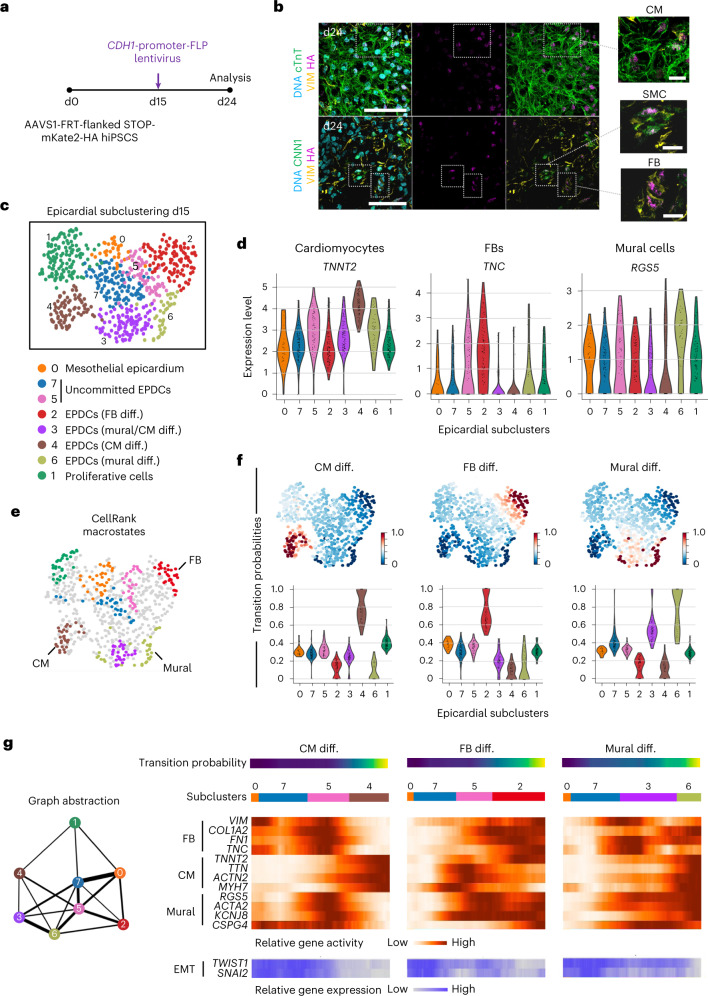
Lineage tracing and multiomic analyses support the trilineage potential of mesothelial epicardial cells in epicardioids. **a**, Schematic of the experimental protocol used for lineage tracing of mesothelial epicardial cells. Epicardioids differentiated from AAVS1-FRT-flanked STOP-mKate2-HA reporter hiPSCs were transduced with a lentiviral vector encoding FLP under the control of the *CDH1* promoter at day 15 before analysis at day 24. **b**, Representative images of immunostaining for the HA tag, the CM marker cTnT and the mesenchymal marker vimentin (top) or the HA tag, vimentin and the SMC marker calponin (CNN1; bottom) in infected organoids at day 24; *n* = 26 epicardioids analyzed (3 to 4 sections each) from 5 independent differentiations, of which 22 contained labeled CMs; scale bars, 50 µm. Insets show exemplary labeled CMs, SMCs and fibroblasts at higher magnification; scale bars, 10 µm. The inset with labeled CMs is shown as a 3D reconstruction in Supplementary Video 3. **c**, UMAP plot showing metacell subclusters of the epicardial lineage at day 15; cell types are annotated. **d**, Violin plots showing the expression levels of markers of CMs (*TNNT2*), fibroblasts (*TNC*) and mural cells (vascular SMCs and pericytes; *RGS5*) in the epicardial subclusters presented in **c**. **e**, UMAP plot showing the macrostates inferred in the epicardial subclustering by CellRank; the most advanced differentiation states are annotated. **f**, UMAP plots (top) and violin plots (bottom) showing the transition probabilities of cells for the fibroblast, CM and mural states inferred by CellRank. **g**, Left, partition-based graph abstraction of the paths taken by cells within the epicardial subclustering. Right, heat maps showing the relative gene activity of fibroblast (*VIM*, *TNC*, *FN1* and *COL1A2*), CM (*TNNT2*, *TTN*, *ACTN2* and *MYH7*) and mural (*RGS5*, *KCNJ8*, *ACTA2* and *CSPG4*) markers (orange) and the relative gene expression of EMT markers (*TWIST1* and *SNAI2*; blue) along the indicated differentiation trajectories.

**Extended Data Fig. 9:**
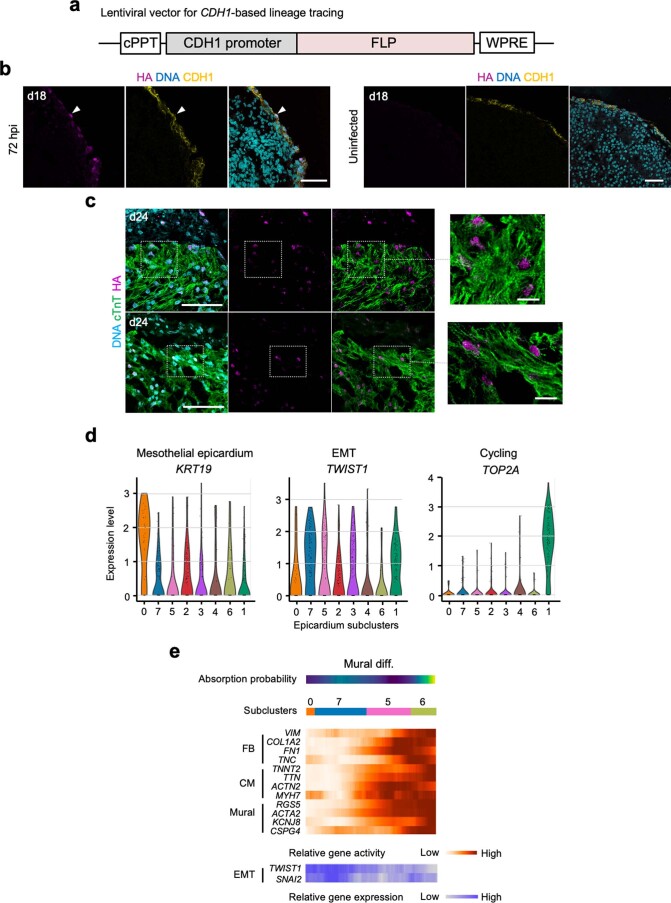
Analysis of epicardial cells and their derivatives by lineage tracing and multi-omic analysis. **(a)** Schematic representation of the lentiviral vector used for lineage tracing of mesothelial epicardial cells in epicardioids derived from AAVS1-CAG-FRT-flanked STOP-mKate2-HA reporter hiPSCs, consisting of the sequence encoding flippase (FLP) driven by the *CDH1* promoter. **(b)** (Left) Immunostaining for the HA-tag and CDH1 in epicardioids at day 18, 72 h after infection with the CDH1-FLP lentivirus. The arrowhead shows an exemplary HA-tag^+^ CDH1^+^ epicardial cell at the outer layer. (Right) Corresponding uninfected negative control. Images are representative of 3 independent differentiations. Scale bars = 50 µm. **(c)** Immunostaining for the HA-tag and cardiac troponin T (cTnT) in infected organoids at day 24, showing HA-labeled cardiomyocytes (CMs) close to the epicardium (representative of 5 independent differentiations). Scale bars = 50 µm. Insets show exemplary labeled CMs at higher magnification; scale bars = 10 µm. **(d)** Violin plots showing the expression levels of markers of mesothelial epicardium (*KRT19*), EMT (*TWIST1*), and cycling cells (*TOP2A*) in epicardial metacell subclusters obtained from analysis of epicardioids by paired scRNA-seq and scATAC-seq. **(e)** Heatmap showing the relative gene activity of FB (*VIM*, *TNC*, *FN1*, *COL1A2*), CM (*TNNT2*, *TTN*, *ACTN2*, *MYH7*) and mural (*RGS5*, *KCNJ8*, *ACTA2, CSPG4*) lineage markers (orange) and the relative gene expression of EMT markers (*TWIST1*, *SNAI2*) (blue) along an alternative mural differentiation trajectory as the one presented in Fig. 5g. CM: cardiomyocyte; FB: fibroblast; EPDC: epicardium-derived cell; EMT: epithelial-to-mesenchymal transition.

To verify the lineage potential of mesothelial epicardial cells in our system, we generated epicardioids from the FLP/FRT-based hiPSC reporter line and transduced them with a lentiviral vector encoding FLP under the control of the *CDH1* promoter at day 15 (Fig. 5a and Extended Data Fig. 9a). After 72 h, we detected HA-tag^+^CDH1^+^ cells, indicating correct labeling of the mesothelial layer (Extended Data Fig. 9b). On day 24, immunofluorescence analysis revealed HA-tag^+^ SMCs and fibroblasts but also CMs (Fig. 5b, Supplementary Video 3 and Extended Data Fig. 9c), supporting the fate potentiality previously inferred from gene expression (Fig. 1h,i).

We then exploited our metacell dataset to investigate the timing of epicardial fate decisions. For this, we performed subclustering of the epicardial lineage at day 15 (differentially expressed genes (DEGs) listed in Supplementary Table 6) and analyzed cell trajectories using CellRank. We detected mesothelial cells (subcluster 0), EPDCs undergoing EMT (subclusters 3, 5 and 7) and EPDCs differentiating into fibroblasts (subcluster 2), mural cells (subcluster 6) or CMs (subcluster 4) as well as proliferative cells (subcluster 1; Fig. 5c,d and Extended Data Fig. 9d). The transition probabilities to each differentiated cell type were balanced among mesothelial cells, suggesting that their fate was not determined before EMT (Fig. 5e,f). EPDCs in subclusters 7 and 5 also appeared to hold trilineage potential, while EPDCs in subcluster 3 were committed toward mural and, to a lesser extent, myocytic differentiation (Fig. 5f). Assessing chromatin accessibility patterns along epicardial differentiation paths showed that the gene activity for CM, fibroblast and mural lineage markers was highest at the end of each respective trajectory, but, importantly, there was also a peak of gene activity for competing lineage markers during and shortly after EMT, suggesting a highly plastic state of EPDCs (Fig. 5g and Extended Data Fig. 9e). Overall, our data do not support the existence of discrete subsets of embryonic epicardial cells restricted to a single lineage before EMT but rather advocate a model of dynamic fate specification over time.

### Epicardioids mimic left ventricular hypertrophy (LVH) and fibrosis

**Fig. 6:**
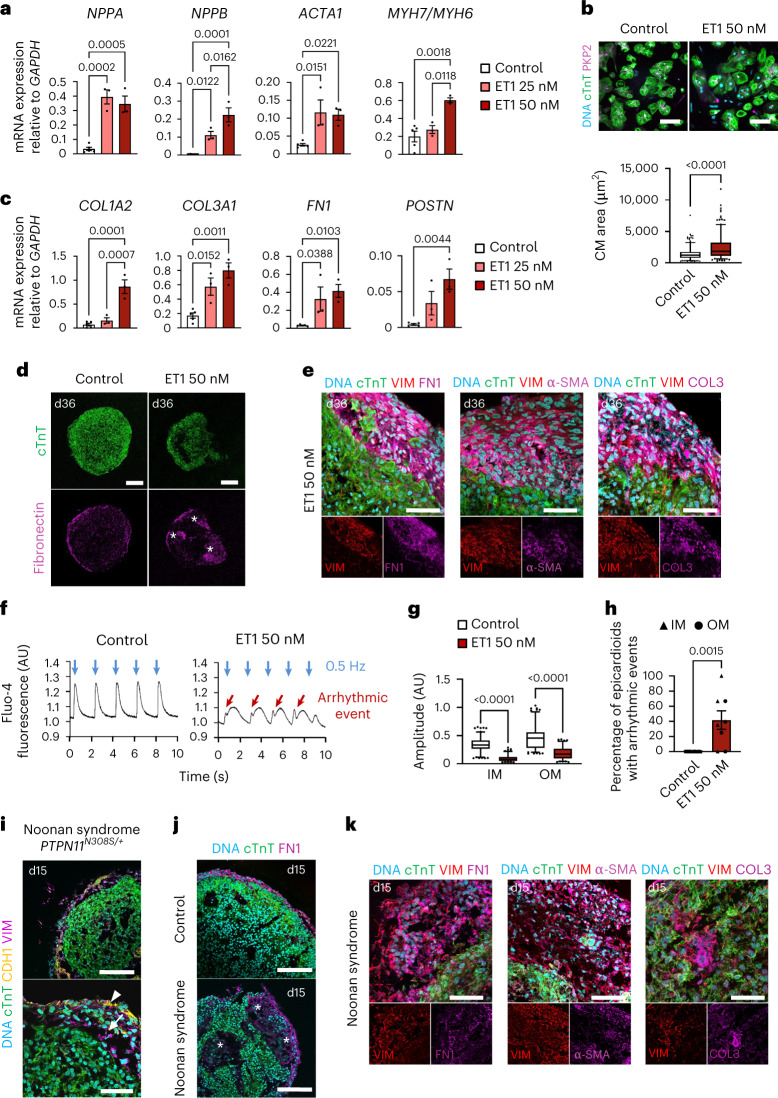
Modeling stress-induced and congenital ventricular hypertrophy and fibrosis in epicardioids. **a**, Expression of hypertrophy markers in ET1-treated epicardioids and controls. Data are shown as mean ± s.e.m.; control, *n* = 5 epicardioids; ET1, *n* = 3 epicardioids per concentration. Two independent differentiations per group were performed. Data were analyzed by one-way ANOVA with a Sidak’s multiple comparisons test. **b**, Top, CMs from day 30 treated or control epicardioids stained for cTnT and plakophilin-2 (PKP2); scale bars, 100 µm. Bottom, CM area; *n* = 260 CMs from 3 differentiations per group. Data were analyzed by unpaired two-tailed *t*-test. **c**, Expression of ECM markers in treated and control epicardioids. Data are shown as mean ± s.e.m.; control, *n* = 5 epicardioids; ET1, *n* = 3 epicardioids per concentration and 2 independent differentiations per group. Data were analyzed by one-way ANOVA with a Sidak’s multiple comparisons test. **d**, Immunostaining for cTnT and FN1 in treated and control epicardioids; scale bars, 200 µm. Asterisks indicate fibrotic remodeling. **e**, Immunostaining for cTnT, α-SMA, FN1 and COL3 in ET1-treated epicardioids; scale bars, 50 µm. **f**, Exemplary Fluo-4 traces in treated and control epicardioids. Blue arrows indicate 0.5-Hz pacing, and red arrows indicate arrhythmic events; AU, arbitrary units. **g**, Calcium transient amplitude in the OM or IM of treated and control epicardioids; control, *n* = 130 transients per layer and 5 epicardioids; ET1, *n* = 106 transients per layer and 4 epicardioids. Three independent differentiations per group were performed. Data were analyzed by Kruskal–Wallis test with a Dunn’s multiple comparisons test. **h**, Percentage of treated and control epicardioids displaying arrhythmic events. Data are shown as mean ± s.e.m.; control, *n* = 5 epicardioids; ET1, *n* = 4 epicardioids. Three independent differentiations per group were performed. Data were analyzed by unpaired two-tailed *t*-test. **i**, Immunostaining for cTnT, CDH1 and vimentin in epicardioids from day 15 hiPSCs derived from an individual with Noonan syndrome; scale bars (top), 100 µm; scale bars (bottom), 50 µm. Arrowheads indicate the mesothelial epicardium layer, and arrows indicate EPDCs. **j**, Immunostaining for cTnT and FN1 in control and Noonan syndrome organoids (day 15). Asterisks indicate fibrotic remodeling; scale bars, 100 µm. **k**, Immunostaining for cTnT, FN1, α-SMA and COL3 in Noonan syndrome epicardioids (day 15); scale bars, 50 µm. Box plots in **b** and **g** indicate the median and 25th and 75th percentiles, with whiskers extending to the 5th and 95th percentiles. Images in **d**, **e** and **i**–**k** are representative of three independent differentiations.

Both inherited and acquired cardiovascular disorders can manifest as LVH, a maladaptive remodeling of the myocardium that increases risk for heart failure and life-threatening arrhythmia^53,54^. Current 2D in vitro models largely recapitulate the myocytic features of LVH but fail to account for the pivotal role of fibrosis in the progression toward heart failure^55,56^. Hypothesizing that the 3D multilineage architecture of epicardioids could resolve this gap, we treated 1-month-old epicardioids with endothelin-1 (ET1), a potent vasoconstrictor known to induce hypertrophy in vivo and in vitro^57,58^. ET1 triggered a dose-dependent upregulation of myocytic hypertrophy markers (*NPPA*, *NPPB*, *ACTA1* and *MYH7*/*MYH6*) and an increase in CM size (Fig. 6a,b). Importantly, the concomitant upregulation of ECM genes (*COL1A2*, *COL3A1*, *FN1* and *POSTN*) suggested the onset of a fibrotic response, which was corroborated by abundant ECM deposition in the subepicardial space and the emergence of α-smooth muscle actin-positive (α-SMA^+^) myofibroblasts (Fig. 6c–e). Calcium imaging in ET1-treated epicardioid slices additionally revealed CM dysfunction across the myocardial layers, including frequent arrhythmic events and decreased calcium transient amplitudes, two well-established features of failing hearts^59^ (Fig. 6f–h).

Having successfully recapitulated a stress-induced phenotype, we next tested the capacity of epicardioids to model congenital myocardial fibrosis. For this, we used hiPSCs from an individual with Noonan syndrome who presented with severe LVH and myocardial fibrosis at birth (Fig. 6i). We recently reported that hiPSC-derived CMs from this individual displayed cell cycle defects, leading to hyperproliferation rather than a classical hypertrophic phenotype when cultured in 2D^60^. The same was observed in individual-specific epicardioids; they did not have larger CMs than healthy controls and did not upregulate hypertrophy markers but showed increased CM proliferation across the myocardial layers (Supplementary Fig. 5a–c). We additionally observed an upregulation of ECM genes and the appearance of areas containing large numbers of fibroblasts and SMA^+^ myofibroblasts as early as day 15, indicating that the cellular environment of epicardioids is indeed permissive to fibrotic changes associated with developmental defects (Fig. 6j,k and Supplementary Fig. 5d).

## Discussion

**Extended Data Fig. 10:**
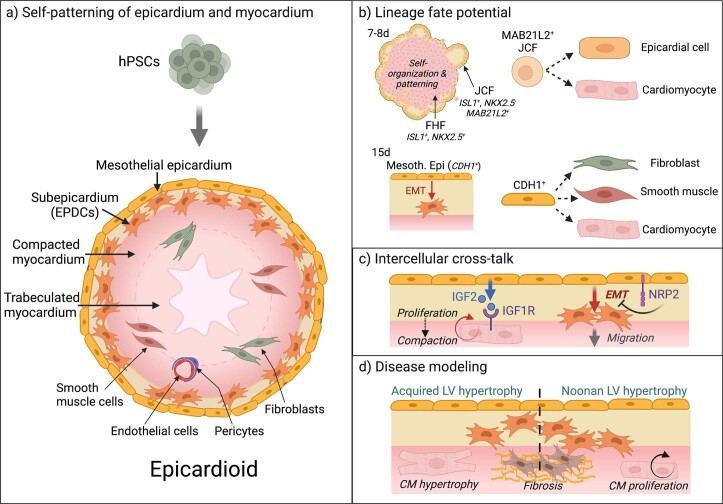
Epicardioids as in vitro models of cardiac development and disease. **(a)** Graphical representation of the structure and cell composition of epicardioids, formed by self-organization of hPSCs into myocardial and epicardial compartments. **(b)** During epicardioid development, juxta-cardiac field (JCF) progenitors give rise to epicardial cells and cardiomyocytes. In turn, mesothelial epicardial cells undergo epithelial-to-mesenchymal transition (EMT) and produce fibroblasts, smooth muscle cells, and cardiomyocytes. **(c)** Epicardioids exhibit functional cross-talk between the epicardial and myocardial compartments. We could demonstrate that IGF2 secreted by the epicardium stimulates cardiomyocyte proliferation and compaction, and that NRP2 regulates epicardial EMT and the migration of epicardium-derived cells. **(d)** Epicardioids are versatile disease models, as illustrated by their capacity to recapitulate hypertrophic and fibrotic remodeling driven by either external stressors (endothelin-1) or congenital defects (Noonan syndrome). Created with BioRender.com.

We have established human epicardioids showing RA-dependent self-organization of ventricular myocardium and epicardium (Extended Data Fig. 10). Epicardioids recapitulate the two major functions of the embryonic epicardium: (1) being the source of progenitors of several cardiac lineages and (2) providing a paracrine milieu driving myocardial compaction and maturation. The latter appears to be key in achieving a high degree of morphological, molecular and functional self-patterning of the myocardium, which has so far been lacking in cardiac organoid models^5,6^. This allowed us to demonstrate that epicardial secretion of IGF2 promotes human myocardial compaction as it does in the mouse^3,33,36^.

Single-cell transcriptomic analyses of human embryonic and adult heart tissue have provided precious insights into epicardial development, which were critical in verifying the validity of our in vitro model. However, isolated tissues represent punctual snapshots that are of limited use for studying dynamic developmental processes, especially those occurring at the earliest stages of cardiogenesis. Epicardioids offer a powerful alternative, as they closely mimic the steps of fetal (left) ventricular development and maturation. Paired transcriptomic and chromatin accessibility profiling in epicardioids notably revealed the existence of a human equivalent of the recently described mouse JCF^44,45^. Conditional lineage tracing based on *MAB21L2* confirmed that human JCF progenitors can give rise to both CMs and epicardial cells, with each fate associated with distinct gene regulatory programs. Our findings suggest that the JCF is not a uniform population regarding lineage potentiality, but further clonal analyses will be required to resolve this aspect and confirm descendance from the pre-JCF precursors identified in our study. We discovered ISL1 as a marker of the human pre-JCF, with maintained expression in the JCF and early epicardium.

Epicardioids also allowed us to address open questions related to epicardial heterogeneity and fate potential. Importantly, lineage tracing of CDH1^+^ mesothelial epicardial cells supported the still-debated myocytic potential of early epicardium, at least in vitro. Moreover, for all three epicardial derivatives (fibroblasts, SMCs and CMs), chromatin accessibility patterns suggested that fate decisions occur after epicardial EMT. This is consistent with recent work in the mouse challenging the long-held notion that there exist distinct epicardial subcompartments^25^.

Finally, we could demonstrate that epicardioids have the unique ability of recapitulating both hypertrophic and fibrotic features of LVH. Epicardioids could therefore be exploited for preclinical testing to identify drugs targeting both aspects of the disease, which are intimately linked during the progression toward heart failure. More broadly, epicardioids offer advantages for modeling complex cardiac disorders, including congenital heart diseases, by allowing dissection of inter- and intracellular cross-talk dynamics during development and disease^61^. We notably discovered a new role of NRP2 signaling in the regulation of epicardial EMT, with potential implications for heart repair^41^. Insights from epicardioids could also lead to new strategies to replace CMs lost during myocardial infarction, arguably one of the biggest challenges of modern medicine, either by reactivating the epicardium’s capacity to promote CM proliferation or by triggering de novo differentiation of EPDCs into CMs. As such, epicardioids offer a unique platform to tackle fundamental questions in developmental biology as well as cardiovascular medicine and drug discovery.

## Methods

### Ethics

This study was approved by the Ethics Commission of the Technical University of Munich Faculty of Medicine (447/17S, 384/15) as part of the European Research Council grant ERC 788381 to A.M. Authorization to use the human embryonic stem cell (hESC) line HES-3 (hPSCreg ESIBIe003) generated by ES Cell International in Singapore was granted by the Central Ethics Committee for Stem Cell Research of the Robert Koch Institute to A.M. (AZ 3.04.02/0131). The Regional Ethical Review Board in Stockholm (Regionala etikprövningsnämnden i Stockholm) approved the study protocol using human aborted embryos with ethical permission number Dnr 2015/1369-31/2 (ref. ^62^). Informed consent was obtained from all donors of cells and tissues.

### Culture of hPSCs

hiPSCs were generated using the CytoTune-iPS 2.9 Sendai reprogramming kit (Invitrogen, A16157) as previously described^63^. The following hiPSC lines were used in differentiation experiments: hPSCreg MRIi003-A (hiPSC1), MRIi001-A (hiPSC2), MRIi003-A-6 (AAVS1-CAG-VSFP; hiPSC3), MRIi003-A-9 (AAVS1-CAG-FRT-flanked STOP-mKate2-HA) and MRIi025-A (*PTPN11*^N308S/+^). The HES-3 line (hPSCreg ESIBIe003; hESC) was generously provided by D. A. Elliott of the Murdoch Children’s Research Institute and Monash Immunology and Stem Cell Laboratories, Monash University^64^. hPSCs were cultured on Geltrex-coated plates (Gibco, A14133-02) in essential 8 medium (Gibco, A1517001) containing 0.5% penicillin/streptomycin (Gibco, 15140-122). Cells were passaged every 4 d with 0.5 mM EDTA (Invitrogen, AM92606) in PBS without Ca^2+^ or Mg^2+^ (PBS^−/−^; Gibco, 10010023).

### Three-dimensional cardiac induction

On day −1, 30,000–40,000 hPSCs were seeded into poly-HEMA-coated (Sigma-Aldrich, P3932) U-shaped 96-well plates in essential 8 medium containing 2 µM thiazovivin. The basal differentiation medium was prepared by mixing 247.36 ml of DMEM/F-12 with GlutaMAX (Gibco, 31331028), 237.36 ml of IMDM (Gibco, 21980032), 5 ml of chemically defined lipid concentrate (Gibco, 11905031), 10 ml of IMDM containing 10% bovine serum albumin (BSA), 250 µl of transferrin (Roche, 10652202001) and 20 µl of α-monothioglycerol (Sigma-Aldrich, M6145). On day 0, essential 8 medium was replaced with basal medium supplemented with 10 ng ml^–1^ BMP4 (R&D, 314-BP), 50 ng ml^–1^ activin A (Sigma-Aldrich, SRP3003), 30 ng ml^–1^ bFGF (R&D, 233-FB-025/CF), 5 µM LY-29004 (Tocris, 1130) and 1.5 µM CHIR-99021 (Axon Medchem, 1386). On day 2, the medium was replaced with basal medium supplemented with 10 µg ml^–1^ insulin (Sigma-Aldrich, I9278), 10 ng ml^–1^ BMP4, 8 ng ml^–1^ bFGF, 5 µM IWP2 (Tocris, 3533) and, where indicated, 0.5 µM RA (Sigma-Aldrich, R2625). This medium was refreshed every 24 h until day 6, at which point the medium was replaced with basal medium supplemented with 10 µg ml^–1^ insulin, 10 ng ml^–1^ BMP4 and 8 ng ml^–1^ bFGF. This medium was refreshed 24 h later on day 7. On day 8, spheroids were embedded in a collagen I solution consisting of 2.17 mg ml^–1^ collagen I (Corning, 354249), 20% distilled water (Gibco, 15230162), 5% 10× DPBS (Gibco, 14080055) and 8.3 mM NaOH freshly added to medium consisting of DMEM/F-12 with 20% fetal bovine serum, 1% non-essential amino acids (Gibco, 11140050), 1% penicillin–streptomycin–glutamine (Gibco, 10378016) and 0.1 mM β-mercaptoethanol (Sigma-Aldrich, M7522). Gel sheets were transferred to maintenance medium consisting of basal medium supplemented with 10 µg ml^–1^ insulin and 0.5% penicillin–streptomycin, and plates were placed on a rocking shaker (Assistant) at 40 r.p.m. Where indicated, 100 ng ml^–1^ VEGF (R&D, 293-VE-010) was freshly added to the medium at each medium change from this point on. For long-term culture, maintenance medium was replaced every 2–3 d.

### Cell culture treatments

In cell–cell interaction experiments, epicardioids were treated with 0.25 µM, 0.5 µM or 1 µM linsitinib (Tocris, 7652) or 200 µg ml^–1^ or 500 µg ml^–1^ NRP2 blocking antibody (R&D, AF2215) in maintenance medium on days 11, 12, 13 and 14. Spheroids differentiated without RA were treated with 25 ng ml^–1^, 50 ng mL^–1^ or 100 ng ml^–1^ recombinant human IGF2 (R&D, 292-G2) in maintenance medium on days 11, 12, 13 and 14. DMSO was used as a vehicle control.

To induce hypertrophy, day 30 epicardioids were treated with 25 nM or 50 nM ET1 (Sigma-Aldrich, E7764) in maintenance medium for 6 d, and the medium was replaced every day. Epicardioids were then either dissociated with papain for reseeding, as described later, dissociated with TrypLE Express (Gibco, 12605010) for 15 min at 37 °C for RNA extraction or fixed.

### Lineage tracing

#### Generation of the AAVS1-CAG-FRT-flanked STOP-mKate2-HA reporter line

To construct the donor plasmid pAAVS1-CAG-FRT-flanked STOP-mKate2-HA-poly(A), the pCAFNF-green fluorescent protein (pCAFNF-GFP) plasmid (Addgene, 13772) was digested with SpeI and SalI, and the CAG-FRT-flanked STOP cassette (CAG promoter and neomycin resistance gene flanked by FRT sites) was cloned into the pAAVS1-Nst-MCS vector (Addgene, 80487), which was digested with SpeI and SalI. The simian virus 40 poly(A) (Sv40-poly(A)) signal was then amplified by PCR from the pCAFNF-GFP plasmid using primers containing Pacl restriction sites at the 5′ end and EcoRI restriction sites at the 3′ end and introduced into the pAAVS1-CAG-FRT-flanked STOP plasmid, digested with PacI and EcoRI. The mKate2 coding sequence fused to an HA tag was amplified by PCR from the p3E-mKate2-HA no-pA plasmid (Addgene, 80810) as a template and inserted into SwaI–PacI sites on the pAAVS1-CAG-FRT-flanked STOP-poly(A) plasmid. Primers used for cloning and sequencing of the pAAVS1-CAG-FRT-flanked STOP-mKate2-HA-poly(A) construct are listed in Supplementary Table 7.

Healthy control hiPSCs (hPSCreg MRI003-A; 1 × 10^6^) were nucleofected with 1 µg of pXAT2 plasmid (Addgene, 80494) containing sequences for an AAVS1 locus-specific single guide RNA (GGG GCC ACT AGG GAC AGG AT) and the Cas9 nuclease and 3 µg of donor construct (pAAVS1-CAG-FRT-flanked STOP-mKate2-HA-poly(A)) following the Lonza Amaxa 4D Nucleofector protocol for human stem cells. Cells were subsequently plated onto Matrigel-coated (BD, 354277) six-well plates (Nunclon, 150687) in mTeSR1 (Stemcell Technologies, 05854) with 10 μM thiazovivin. Twenty-four hours later, and every day afterward, the medium was replaced with fresh mTeSR1. Three days after nucleofection, 150 μg ml^–1^ neomycin (Gibco, 10131) was added into the mTeSR1 for selection for 2 weeks. When the hiPSC colonies were large enough, cells were dissociated with Accutase (Thermo Fisher Scientific, A11105-01) and replated for single-clone expansion at low density (1,000 cells per 10-cm Matrigel-coated dish). Single clones were then picked for PCR genotyping and further expansion into wells of a Matrigel-coated 96-well plate (Nunclon, 161093). The genotype of the selected clones was verified by PCR screening and confirmed by Sanger sequencing (Eurofins MWG Operon; primers listed in Supplementary Table 7).

Karyotype analysis after editing was performed at the Institute of Human Genetics of the Technical University of Munich using G-banding (20 metaphases counted). Three of ten potential off-target sites predicted by the CRISPOR tool (https://crispor.tefor.net) were amplified and verified by Sanger sequencing (primers are listed in Supplementary Table 7). To verify correct reporter expression, positive hiPSCs clones (1 × 10^6^) were nucleofected with 3 µg of pCAGGS T2A FLPo plasmid (containing the coding sequence of puromycin in frame with FLPo; Addgene, 124835) and kept in culture as described above. Three days after nucleofection, antibiotic selection with 0.2 μg ml^–1^ puromycin (Calbiochem, 540411) was induced for 10 d. Cells were then fixed and immunostained with anti-HA tag as described later (antibodies are listed in Supplementary Tables 8 and 9).

#### Generation of lentiviral *CDH1* and *MAB21L2* promoter reporter constructs and lineage tracing of JCF and mesothelial epicardium

For the generation of the lentiviral transfer vector carrying an FLP under control of the human ∼1.37-kilobase (kb) *CDH1* promoter, red fluorescent protein (RFP) from the lentiviral pHAGE-E-cadherin-promoter-RFP plasmid (Addgene, 79603) was replaced by an FLP from the plasmid pCAGS-T2A-FLP (Addgene, 123845). Lentiviral transfer vectors carrying a tamoxifen-inducible FLP under the control of the human ∼1.88-kb *MAB21L2* promoter (chromosome 4: 150581151–150583029) were synthetized by Vectorbuilder.

Lentiviruses were produced in HEK293T cells by transient cotransfection of the lentiviral transfer vector, the CMVDR8.74 packaging plasmid and the VGV.G envelope plasmid using Fugene HD (Promega, E2311). Viral supernatants were collected after 48 h and used for infection of epicardioids derived from the AAVS1-CAG-FRT-flanked STOP-mKate2-HA reporter hiPSCs in the presence of 8 µg ml^–1^ polybrene (Sigma-Aldrich, 107689).

For lineage tracing of JCF cells, epicardioids were infected at day 3 with the *MAB21L2*-promoter-FLP^ERT2^ lentivirus, and 2.5 µM 4-OHT (Sigma-Aldrich, H6278) was applied at days 4 and 5 or days 7 and 8 to induce FLP expression. Epicardioids were then collected at day 8 or day 12 for immunofluorescence analysis. For lineage tracing of mesothelial epicardial cells, epicardioids were infected at day 15 with the *CDH1*-promoter-FLP lentivirus and collected at day 18 or day 24 for immunofluorescence analysis.

### Immunofluorescence analysis

Cryosections of spheroids were prepared as described by Lancaster and Knoblich, with some modifications^65^. Briefly, spheroids were washed with DPBS and fixed with 4% paraformaldehyde (Sigma-Aldrich, 158127) for 1 h at room temperature. After washing three times with DPBS, spheroids were kept in 30% sucrose at 4 °C overnight and embedded in a solution of 10% sucrose and 7.5% gelatin in DPBS before freezing in a 2-methyl-butane bath (Sigma-Aldrich, M32631) cooled with liquid nitrogen and transferring to −80 °C. Cryosections prepared with a Microm HM 560 cryostat (Thermo Fisher Scientific) were transferred onto poly-l-lysine slides (Thermo Fisher Scientific, J2800AMNT) and stored at −80 °C.

For immunostaining, samples were washed with DPBS and fixed with 4% paraformaldehyde at room temperature for 15 min (cells) or 10 min (cryosections). After washing three times with DPBS, samples were permeabilized with 0.25% Triton X-100 (Sigma-Aldrich, X100) in DPBS for 15 min at room temperature. After washing another three times with DPBS, samples were blocked with 3% BSA in DPBS + 0.05% Tween 20 (PBST; Sigma-Aldrich, P2287) for 1 h at room temperature. Primary antibodies (Supplementary Table 8) were then added at the indicated dilutions in 0.5% BSA in PBST and incubated overnight at 4 °C. After washing three times for 5 min (cells) or five times for 10 min (cryosections) with PBST, appropriate secondary antibodies (Supplementary Table 9) diluted 1:500 in 0.5% BSA (Sigma-Aldrich, A9647) in PBST were added for 1 h (cells) or 2 h (cryosections) at room temperature protected from light. After repeating the previous washing steps, Hoechst 33258 (Sigma-Aldrich, 94403) was added at a final concentration of 5 µg ml^–1^ in DPBS for 15 min at room temperature protected from light. Samples were mounted with fluorescence mounting medium (Dako, S3023) and stored at 4 °C until imaging with an inverted or confocal laser-scanning microscope (DMI6000B and TCS SP8, Leica Microsystems). Images were acquired and processed using the Leica Application Suite X software (v3.5.7.23225).

### Cell preparation for single-cell sequencing

Epicardioids were dissociated to single cells using papain, as previously described^66^, by adapting the number of pooled epicardioids and dissociation time to the stage of development (Supplementary Table 10). Briefly, a 2× papain solution consisting of 40 U ml^–1^ papain (Worthington Biochemical, LS003124) and 2 mM l-cysteine (Sigma-Aldrich, C6852) in PBS^−/−^ was incubated for 10 min at 37 °C to activate the papain before diluting 1:2 in PBS^−/−^ to obtain the 1× solution. Spheroids were then removed from the collagen gel if necessary and washed twice with 2 mM EDTA in PBS^−/−^. Spheroids were then dissociated in 750 µl of 1× papain solution at 37 °C and 750 r.p.m. on a thermomixer (Eppendorf). The enzymatic reaction was stopped with 750 µl of stop solution consisting of 1 mg ml^–1^ trypsin inhibitor (Sigma-Aldrich, T9253) in PBS^−/−^. After pipetting up and down approximately 30 times to obtain a single-cell suspension, cells were passed through a 40-µm strainer and washed with 5 ml of 1% BSA (Gibco, 15260037) in PBS^−/−^. After centrifugation for 3 min at 200*g*, cells were resuspended in 500 µl of 0.5% BSA in PBS^−/−^ for counting with trypan blue. For samples exceeding 15% cell death, dead cells were immediately depleted using a dead cell removal kit (Miltenyi Biotec, 130-090-101), according to the manufacturer’s instructions, before further processing. Cells from the same cell suspension were then used for scRNA-seq and scATAC-seq as described below.

### scRNA-seq

After dissociation, samples were processed for scRNA-seq with a targeted cell recovery of 8,000. To generate Gel Bead-In-EMulsions (GEMs) and single-cell sequencing libraries, the Chromium Single Cell 3′ GEM Library & Gel Bead kit v3 (10x Genomics, 1000092), Chromium Chip B Single Cell kit (10x Genomics, 1000073) and Chromium i7 Multiplex kit v2 (10x Genomics, 120262) were used for samples from days 2 to 15, and the Chromium Next GEM Single Cell 3′ Library & Gel Bead kit v3.1 (10x Genomics, 1000128), Chromium Single Cell G Chip kit (10x Genomics, 1000127) and Single Index kit T set A (10x Genomics, 1000213) were used for the day 30 sample. Quality control of cDNA samples was performed on a Bioanalyzer (Agilent) using a high-sensitivity DNA kit (Agilent, 5067-4626). Library quantification was performed with the KAPA quantification kit (KAPA Biosystems, KK4824) following the manufacturer’s instructions. Libraries were pooled and sequenced using a NovaSeq S1 flow cell (Illumina) with 150-base pair (bp) paired-end reads with 28 cycles for read 1, 91 cycles for read 2, 8 cycles for i7 and 0 cycles for i5 and with a read depth of at least 25,000–30,000 paired-end reads per cell.

The Cell Ranger pipeline (v6.1.1) was used to perform sample demultiplexing and barcode processing and to generate the single-cell gene counting matrix. Briefly, samples were demultiplexed to produce a pair of FASTQ files for each sample. Reads containing sequence information were aligned using the reference provided with Cell Ranger (v6.1.1) based on the GRCh37 reference genome and ENSEMBL gene annotation. PCR duplicates were removed by matching the same unique molecular identifier (UMI), 10x barcode and gene and collapsing them to a single UMI count in the gene–barcode UMI count matrix. All the samples were aggregated using Cell Ranger with no normalization and treated as a single dataset. The R statistical programming language (v3.5.1) was used for further analysis.

The count data matrix was read into R and used to construct a Seurat object (v4.1.1). The Seurat package was used to produce diagnostic quality control plots and to select thresholds for further filtering. Filtering method was used to detect outliers and high numbers of mitochondrial transcripts. These preprocessed data were then analyzed to identify variable genes, which were used to perform a principal-component analysis (PCA). Statistically significant PCs were selected by PC elbow plots and used for uniform manifold approximation and projection (UMAP) analysis. Clustering parameter resolution was set to 1 for the function FindClusters() in Seurat. For subclustering analysis, we used the clustree package (v0.4.3). All DEGs were obtained using a Wilcoxon rank-sum test using as threshold *P* value of ≤0.05. We used adjusted *P* values based on Bonferroni correction using all features in the dataset. For cell-type-specific analyses, single cells of each cell type were identified using the FindConservedMarkers function, as described within the Seurat pipeline. Cellular dynamics were inferred based on the kinetics of gene expression using RNA velocity^21^. Analysis of cell–cell interactions was performed with CellPhoneDB v2.1.7 (ref. ^30^). For all the gene signatures analyzed, we used a function implemented in the yaGST R package v2017.08.25 (https://rdrr.io/github/miccec/yaGST/)^67^.

For analysis of the 2D epicardium scRNA-seq dataset from Gambardella et al.^11^, we downloaded the raw data from https://www.ncbi.nlm.nih.gov/geo/query/acc.cgi?acc=GSE122827. Reads containing sequence information were aligned using the GRCh37 reference genome and ENSEMBL gene annotation, as used for the data generated in our study. The Seurat pipeline (v4.0.1) was used to produce diagnostic quality control plots and to select thresholds for further filtering to get the UMAP plot presented in Extended Data Fig. 6a.

To compare our dataset from day 15 and day 30 with a published scRNA-seq dataset of human embryonic heart development^18^, we downloaded the UMI counts of the Cui et al. dataset from https://www.ncbi.nlm.nih.gov/geo/query/acc.cgi?acc=GSE106118. Identification of common genes between the Cui et al. dataset and ours was based on *Homo sapiens* gene symbols. Filtering of the data and annotating cell types were performed based on cell identity information provided in ref. ^18^. For the earliest epicardial population (referred to as proepicardial), no unique identifier was provided, and these cells were identified based on a de novo clustering of the Cui et al. dataset (Seurat pipeline with *t*-distributed stochastic neighbor embedding and standard settings), which allowed the identification of a distinct cluster of cells from the 5-week time point corresponding to the proepicardial transcriptional profile described in their manuscript. For correlation analysis, cell-type-specific genes were selected through differential expression analysis between the various cell types in the Cui et al. dataset (top 30 with the lowest adjusted *P* value; data were analyzed by Wilcoxon rank-sum test; adjusted *P* value of <0.01). We calculated the average log-normalized expression values for each cluster of the day 30 dataset and the various cell types of the Cui et al. dataset and then computed the Pearson correlation based on the above-mentioned cell-type-specific markers with the function cor() of the R package stats version 4.2.2. The results were plotted as a heat map showing Pearson correlation coefficients in pseudocolor.

### scATAC-seq

After dissociation, nuclei isolation for scATAC-seq was performed following the recommendations of 10x Genomics. Briefly, ~500,000 cells from each sample were transferred to a 1.5-ml microcentrifuge tube and centrifuged at 300*g* for 5 min at 4 °C. The supernatant was removed without disrupting the cell pellet, and 100 μl of chilled lysis buffer (10 mM Tris-HCl pH 7.4, 10 mM NaCl, 3 mM MgCl_2_, 0.1% Tween 20, 0.01% NP-40 substitute, 0.01% digitonin and 1% BSA) was added and mixed by pipetting ten times. Samples were then incubated on ice for 30–120 s (the optimal incubation time was optimized in advance for each time point). Following lysis, 1 ml of chilled wash buffer (10 mM Tris-HCl pH 7.4, 10 mM NaCl, 3 mM MgCl_2_, 0.1% Tween 20 and 1% BSA) was added and mixed by pipetting. Nuclei were centrifuged at 500*g* for 5 min at 4 °C, the supernatant was removed without disrupting the pellet, and nuclei were resuspended in the appropriate volume of chilled diluted nuclei buffer (10x Genomics) to obtain a nuclei concentration suitable for a target nuclei recovery of 8,000.

Samples were then processed using the Chromium Next Single Cell ATAC Library & Gel Bead kit v1.1 (10x Genomics, 1000175), Chromium Single Cell H Chip kit (10x Genomics, 1000161) and Chromium Single Index kit N, set A (10x Genomics, 1000212) to generate GEMs and scATAC-seq libraries. Libraries were pooled and sequenced using a NovaSeq S1 flow cell (Illumina) with 150-bp paired-end reads with 50 cycles for reads 1 and 2, 8 cycles for i7 and 16 cycles for i5 and with a read depth of at least 25,000–30,000 paired-end reads per cell.

Sequencing raw data were processed using 10x Genomics Cell Ranger ATAC 1.2.0. Before alignment to the human reference genome, the ATAC-seq sequences were quality checked using FastQC. The parameters evaluated were (1) total number of reads, (2) sequencing length distribution, (3) sequence quality per base and (4) duplication level. Metrics were homogeneous among all samples (on average) with more than 91% with a *Q* score of ≥30 and percent duplicates of ≤15%. All samples were aggregated, and joint peak calling was performed using Cell Ranger ATAC aggr with no normalization.

R (v4.1.3) was used for further analysis of the count matrices using Signac^68^ (v1.7.0) and Seurat^69^ (v4.1.1). Quality control metrics (total number of fragments, transcription start site (TSS) enrichment score, nucleosome signal, the percentage of reads in peaks and the ratio of reads in genomic blacklist regions) were computed using Signac. Cells were filtered based on the following cutoffs: total number of fragments between 1,000 and 100,000 fragments per cell, TSS enrichment score between 2 and 10, nucleosome signal of <10, fraction of reads in peaks of >0.2 and blacklist ratio of <0.015. Doublets were detected and filtered out using AMULET^70^ v1.1, which finds cells that have significantly more regions with more than two aligned reads in one position than expected across the genome.

For downstream analysis, peak counts were normalized using the term frequency-inverse document frequency (tf-idf). Gene activities were calculated from the scATAC-seq data using Signac and log normalized with a normalization factor of 10,000.

### Multiomic analyses

#### Integration of scRNA-seq and scATAC-seq data

The unmatched modalities were integrated using GLUE^46^ v0.2.3. The RNA modality input was preprocessed by first selecting the top 2,000 highly variable genes using scanpy^71^ (v1.9.1) with flavor ‘seurat_v3’. The features were then log normalized, and dimensionality reduction was performed using a PCA with 100 components. The PCA embedding was used as a first encoder transformation of the model. For the ATAC modality, we applied latent semantic indexing for dimension reduction as implemented in GLUE. GLUE takes a guidance graph as input that links both modalities. We used the default implementation that links an ATAC peak to a gene if it overlaps either the gene body or promoter region.

To match cells from both modalities, we performed minimum cost maximum flow bipartite matching on the joint embedding derived from GLUE as described and used previously^50,72^. The cost graph was inferred using get_cost_knn_graph with knn_k = 15, null_cost_percentile=99 and capacity_method = ‘uniform’. Using the bipartite matches, we matched each ATAC cell to an RNA cell. In cases where no ATAC match was found for an RNA cell, we used only the RNA information. The latent vector of the cell was calculated as the average latent vector of the matched cells. Gene activities were further denoised with MAGIC^73^ by smoothing over nearby cells in the joint embedding as proposed and benchmarked in ArchR^74^. The Python implementation of magic (v3.0.0) was used to smooth gene activities over the *k*-nearest neighbors graph of the joint embedding with *k* = 15 neighbors, decay = 1 and *k*-nearest neighbors autotune parameter ka = 4.

#### Clustering, DEGs and visualization

Leiden clustering^75^ was performed on the 15-nearest-neighbor graph that was calculated on the latent embedding from GLUE. We used the scanpy^71^ (v1.9.1) function scanpy.tl.leiden with the resolution set to 1. All DEGs were obtained with the Wilcoxon rank-sum test (scanpy.tl.rank_genes_groups) and corrected for multiple testing using the Benjamini–Hochberg method. We applied a significance threshold of 0.05 to the false discovery rate (FDR)-adjusted *P* values. For visualization, a 2D UMAP^76^ of integrated latent space was generated based on the 15-nearest-neighbor graph.

#### Inference of cell fate trajectories

Loom files containing raw spliced and unspliced counts were obtained by running the velocyto command line tool^21^. RNA velocity was calculated on the spliced and unspliced reads of the metacells using scVelo (v0.2.4)^77^. Moments were computed on the 2,000 highly variable features. The RNA velocity was inferred using the function scvelo.tl.velocity with mode = ‘dynamical’. Palantir^78^ was used with the default parameters to infer a pseudotime on the integrated dataset. The root cell was chosen based on the diffusion coefficient. We then used CellRank^47^ (v1.5.1) to compute lineages and absorption probabilities into terminal cell states. The transition matrix was constructed by combining a velocity kernel and a pseudotime kernel with weights of 0.3 and 0.7, respectively, to mainly capture the joint pseudotime. Terminal states were inferred using the compute_macrostates function with n_states = 15. Absorption probabilities for each of the terminal states were computed with the GPCCA estimator.

#### GRN inference

We constructed a GRN for JCF cells using Pando^50^ (v1.0.1). Pando takes the integrated metacells with RNA and ATAC measurements and constructs a GRN based on four main steps^50^:

1. Filtering for candidate regulatory genomic regions.
2. Scanning regions for TF binding motifs.
3. Creating region–TF pairs for each target gene.
4. Inferring relevant TF–region interactions by fitting a regression model with region–TF pairs as variables to predict the expression of the target gene.

We only included peak regions that overlap with PhastCons conserved elements^79^ from the alignment of 30 mammals using the Pando function initiate_grn. The conserved elements are already included in Pando, and we lifted them to the hg19 reference genome using the R package liftOver (v1.18.0). Pando contains a curated motif database that consists of binding motifs from JASPAR (2020 release)^80^ extended by motifs from the CIS-BP database^81^. We considered all TFs and their motifs that were found in the top 4,000 highly variable genes to be relevant. Subsequently, selected peak regions were scanned for motifs using the Pando function find_motifs. We then used the Pando function infer_grn to fit a linear model for each target gene to infer interactions between TF binding site pairs and the gene. TF binding sites in peak regions were considered for a target gene if they overlapped the gene body or 100 kb upstream of the TSS.

#### Gene module construction

The inferred network was further pruned using the Pando function find_modules. Briefly, Pando assesses significance of the inferred coefficients using analysis of variance (ANOVA) and corrects for multiple testing using the Benjamini–Hochberg method. We applied a significance threshold of 0.05 to the FDR-adjusted *P* values. The inferred connections to target genes were then summarized into positive and negative modules of a TF. The module activity of a TF can be represented by the expression of the set of target genes that it regulates. We calculated the gene module activity with the Seurat function AddModuleScore with all genes included in the Pando model as the set of background genes.

#### Visualization of GRN

The GRN was visualized using the Pando function get_network_graph and plot_network_graph with the option umap_method = ‘weighted’, which computes a UMAP embedding of the TFs in the graph based on coexpression and regulatory relationship as measured by the inferred coefficients. Nodes are sized by the PageRank centrality of each TF. To determine whether a TF is more important for the epicardial or the CM lineage, we computed an absorption probability weighted expression^50^. Specifically, we multiplied the *z*-scaled epicardial absorption probability by the expression of a TF in each gene and formed the average over all cells. This way, TFs that show a strong expression correlation with the epicardial absorption probabilities will have a positive weighted expression, while TFs that correlate with the CM lineage will have a negative weighted expression.

#### Branch-specific TF activity

We first clustered the JCF cell population into cells with more epicardial and more CM potential based on our previous CellRank analysis results. Using the absorption probabilities into both fates as features, we applied *k*-means clustering as implemented in the scikit-learn package (v1.1.1) with *k* = 2. Branch-specific TF activity was defined as the product of the mean TF expression per branch and Pando coefficient for all downstream targets.

#### Subclustering of epicardial cells

To determine different lineages in the epicardial cells, we filtered all cells in clusters 17 and 14 originating from day 15 and recomputed the neighborhood graph on the metacell embedding with n_neighbors = 15. Leiden clustering^75^ was performed with a resolution of 0.7. We again used CellRank^47^ (v1.5.1) to get a more fine-grained set of terminal states. As for the inference of cell fate trajectories, the transition matrix was constructed by combining a velocity kernel and a pseudotime kernel with weights of 0.3 and 0.7, respectively. We used partition-based graph abstraction^82^ to infer the connectivity of the inferred clusters. The graph was further pruned to only contain edges with a connectivity score of >0.2. Imputed gene activities and gene expression were visualized along paths in the abstracted graph using the function scanpy.pl.paga_path.

### Vibratome sectioning

To prepare live sections, spheroids were removed from the collagen gel and placed in 4% agarose (Biozym, 840004) in sterile DPBS^+/+^. Once the agarose had solidified, it was trimmed down to a block of approximately 1 cm × 1 cm × 1 cm with a scalpel, and 250-µm-thick slices were cut with a vibratome (VT1200S, Leica Biosystems) in a DPBS bath, following the manufacturer’s instructions. The spheroid slices were then kept in maintenance medium for 3–5 d before functional assays.

### Optical action potential measurements

For optical action potential measurements, 250-µm-thick slices of spheroids derived from the AAVS1-CAG-VSFP hiPSC line^29^ (hPSCreg MRI003-A-6) were transferred to Tyrode’s solution (135 mM NaCl, 5.4 mM KCl, 1 mM MgCl_2_, 10 mM glucose, 1.8 mM CaCl_2_ and 10 mM HEPES, pH 7.35) before imaging at 100 fps on an inverted epifluorescence microscope (DMI6000B, Leica Microsystems) equipped with a Zyla V sCMOS camera (Andor Technology). The VSFP was excited at 480 nm, and the emitted GFP and RFP fluorescence signals were separated using an image splitter (OptoSplit II, Caim Research). The fluorescence of regions of interest relative to background regions was quantified in ImageJ (National Institutes of Health), and subsequent analysis was performed in RStudio^83^ using custom-written scripts to determine the duration at 50% (APD_50_) or 90% repolarization (APD_90_). APD_50_ maps were generated by aligning the split image stacks with a custom algorithm in MatLab (The MathWorks), denoising them with the CANDLE algorithm^84^ and calculating the ratio between the two. For each action potential, the APD was calculated directly based on the amplitude on each pixel.

### Calcium imaging

Calcium imaging was performed as previously described, with some modifications^85^. Briefly, 250-µm-thick spheroid slices were loaded with 1 µM Fluo-4-AM (Thermo Fisher Scientific, F14201) in Tyrode’s solution (135 mM NaCl, 5.4 mM KCl, 1 mM MgCl_2_, 10 mM glucose, 1.8 mM CaCl_2_ and 10 mM HEPES, pH 7.35) containing 0.01% Pluronic F-68 (Gibco, 24040-032) for 50 min at 37 °C. The solution was replaced with Tyrode’s solution for 30 min at 37 °C for deesterification of the dye before imaging at 100 fps on an inverted epifluorescence microscope (DMI6000B, Leica Microsystems) equipped with a Zyla V sCMOS camera (Andor Technology). Pacing was performed with field stimulation electrodes (RC-37FS, Warner Instruments) connected to a stimulus generator (HSE Stimulator P, Hugo-Sachs Elektronik) providing depolarizing pulses at the indicated frequencies. The fluorescence of regions of interest relative to background regions was quantified in ImageJ (National Institutes of Health), and subsequent analysis was performed in RStudio^83^ using custom-written scripts to determine the transient duration at 50% or 90% decay.

### Quantitative real-time PCR

Total RNA was isolated from cells using the Absolutely RNA Microprep kit (Agilent, 400805), and cDNA was prepared using the high-capacity cDNA RT kit (Applied Biosystems, 4368814) according to the manufacturers’ instructions. Quantitative real-time PCR was performed using Power SYBR Green PCR master mix (Applied Biosystems, 4368706; primers are listed in Supplementary Table 11) on a 7500 Fast real-time PCR instrument (Applied Biosystems). The mRNA expression levels of genes of interest were quantified relative to *GAPDH* expression using the cycling threshold (Δ*C*_*t*_) method.

### Measurement of CM size

For cell size measurements, epicardioids were dissociated to single cells with papain as described above and reseeded at a density of 25,000 cells per cm^2^ on plates coated with 2 µg cm^–2^ fibronectin (Sigma-Aldrich, F1141). After 4 d, cells were fixed for immunofluorescence staining for cTnT and the desmosomal marker plakophilin-2 to visualize cell membranes, as described above. The area of CMs was quantified in ImageJ (National Institutes of Health).

### Statistics

Statistical analysis was performed with GraphPad Prism version 9.1.0. Box-and-whiskers plots indicate the median and 25th and 75th percentiles, with whiskers extending to the 5th and 95th percentiles; bar graphs indicate the mean ± s.e.m. of all data points, unless otherwise indicated. Normally distributed data from two experimental groups were compared by Student’s *t-*test; otherwise a Mann–Whitney–Wilcoxon test was applied. Normally distributed data from more than two experimental groups were compared using one- or two-way ANOVA. In the case of multiple comparisons, an appropriate post hoc test was applied as indicated. A *P* value of <0.05 was considered statistically significant.

### Reporting summary

Further information on research design is available in the Nature Portfolio Reporting Summary linked to this article.

## Online content

Any methods, additional references, Nature Portfolio reporting summaries, source data, extended data, supplementary information, acknowledgements, peer review information; details of author contributions and competing interests; and statements of data and code availability are available at 10.1038/s41587-023-01718-7.

### Supplementary information

Supplementary Information Supplementary Results, Figs. 1–5 and Tables 7–11.

Reporting Summary

Supplementary Video 1 Brightfield recording of a beating day 15 spheroid differentiated with RA.

Supplementary Video 2 Brightfield recording of a beating day 15 spheroid differentiated without RA.

Supplementary Video 3 Immunostained section of an epicardioid differentiated from AAVS1-FRT-flanked STOP-mKate2-HA reporter hiPSCs and transduced with a lentiviral vector encoding FLP under the control of the *CDH1* promoter at day 15 before analysis at day 24. Cells derived from *CDH1*^+^ epicardial cells are marked by the HA tag in magenta, cardiomyocytes are marked by cTnT in green, mesenchymal cells are marked by vimentin in yellow, and nuclei are counterstained with Hoechst in cyan; scale bar, 20 µm. This area corresponds to the image shown in Fig. 5b.

Supplementary Tables 1–6 Supplementary Table 1. DEGs of the 14 clusters obtained from scRNA-seq analysis of epicardioids day 15 and day 30. DEGs were obtained using a Wilcoxon rank-sum test using as threshold *P* value of <0.05. We used adjusted *P* values based on Bonferroni correction using all features in the dataset. Supplementary Table 2. DEGs of the 11 epicardial subclusters in epicardioids day 15 and day 30. DEGs were obtained using a Wilcoxon rank-sum test using as threshold *P* value of <0.05. We used adjusted *P* values based on Bonferroni correction using all features in the dataset. Supplementary Table 3. DEGs of the 24 metacell clusters obtained from paired scRNA-seq and scATAC-seq analysis of epicardioids at days 2, 3, 4, 5, 7, 10 and 15. DEGs were obtained with the Wilcoxon rank-sum test (scanpy.tl.rank_genes_groups) and corrected for multiple testing using the Benjamini–Hochberg method. We applied a significance threshold of 0.05 to the FDR-adjusted *P* values. Supplementary Table 4. Positive downstream targets of TFs in the GRN of the metacell cluster 14 (JCF). Supplementary Table 5. Negative downstream targets of TFs in the GRN of the metacell cluster 14 (JCF). Supplementary Table 6. DEGs of the seven epicardial metacell subclusters in epicardioids day 15. DEGs were obtained with the Wilcoxon rank-sum test (scanpy.tl.rank_genes_groups) and corrected for multiple testing using the Benjamini–Hochberg method. We applied a significance threshold of 0.05 to the FDR-adjusted *P* values.

Supplementary Data Statistical source data for Supplementary Figs. 1 and 5.

Supplementary Data Uncropped scans of the PCR gels presented in Supplementary Fig. 4b.

### Source data

Source Data Figs. 2, 3 and 6 and Extended Data Figs. 1, 2, 5 and 6 Statistical source data for Figs. 2, 3 and 6 and Extended Data Figs. 1, 2, 5 and 6.

## Data Availability

All scRNA-seq and scATAC-seq data that support the findings of this study can be found at Gene Expression Omnibus under the accession number GSE196516 (ref. ^86^). Reads containing sequence information were aligned using the GRCh37 reference genome and ENSEMBL gene annotation (http://igenomes.illumina.com.s3-website-us-east-1.amazonaws.com/Homo_sapiens/Ensembl/GRCh37/Homo_sapiens_Ensembl_GRCh37.tar.gz). For analysis of the 2D epicardium scRNA-seq dataset from Gambardella et al.^11^, we downloaded the raw data from accession ID GSE122827. We downloaded the UMI counts of the Cui et al.^18^ dataset from accession ID GSE106118. Source data are provided with this paper. Any other data supporting the findings of this study are available from the corresponding author on reasonable request.

## Code availability

Jupyter notebooks to reproduce the metacell analysis and figures are available at https://github.com/theislab/epicardiods_analysis.
