# Supplementary material for: Epicardioid single-cell genomics uncovers principles of human epicardium biology in heart development and disease
Source: Nat Biotechnol. 2023 Apr 3;41(12):1787–800. doi: 10.1038/s41587-023-01718-7 (PMC10713454; doi:10.1038/s41587-023-01718-7)
Supplement: Supplementary file 1 — Supplementary Results, Figs. 1–5 and Tables 7–11. [file 41587_2023_1718_MOESM1_ESM.pdf]

nature biotechnology

Article

https://doi.org/10.1038/s41587-023-01718-7

Epicardioid single-cell genomics uncovers principles of human epicardium biology in heart development and disease

In the format provided by the authors and unedited

Supplementary Results

Modulation of RA signaling during 3D cardiac induction

Considering the major role of RA in the formation of epicardioids, we tested the effects of modulating RA timing and dosage during differentiation. Limiting RA exposure to days 2 and 3 of differentiation led to larger, looser spheroids that rarely formed an epicardial layer (Supplementary Fig. 1a-d). By contrast, increasing the concentration of RA to 1 µM on days 2 to 5 resulted in smaller spheroids and slightly reduced the efficiency of epicardium formation (Supplementary Fig. 1a-d). This was reflected in the expression levels of epicardial markers (*BNC1*, *TBX18*, *WT1*) on day 15, which were highest when applying 0.5 µM RA on days 2 to 5 (Supplementary Fig. 1e).

Generation and validation of the AAVS1-CAG-FRT-flanked STOP-mKate2-HA hiPSC line

We used CRISPR/Cas9 gene editing to generate a hiPSC reporter line based on the flippase-mediated recombination system (Flp/FRT). A reporting construct consisting of an FRT-flanked neomycin cassette blocking transcription of a pCAG-driven fluorescent reporter (mKate2) fused to an HA-tag was knocked into the AAVS1 safe harbor locus[49] (Supplementary Fig. 4a,b). After editing, we verified that the hiPSCs maintained a normal karyotype (Supplementary Fig. 4c) and did not carry mutations in the top predicted off-target sites (Supplementary Fig. 4d). Reporter functionality was confirmed by the detection of mKate2 and the HA-tag following transfection with a vector encoding flippase (Supplementary Fig. 4e,f). After validation, the reporter line was entered into the Human Pluripotent Stem Cell Registry under the ID MRIi003-A-9.

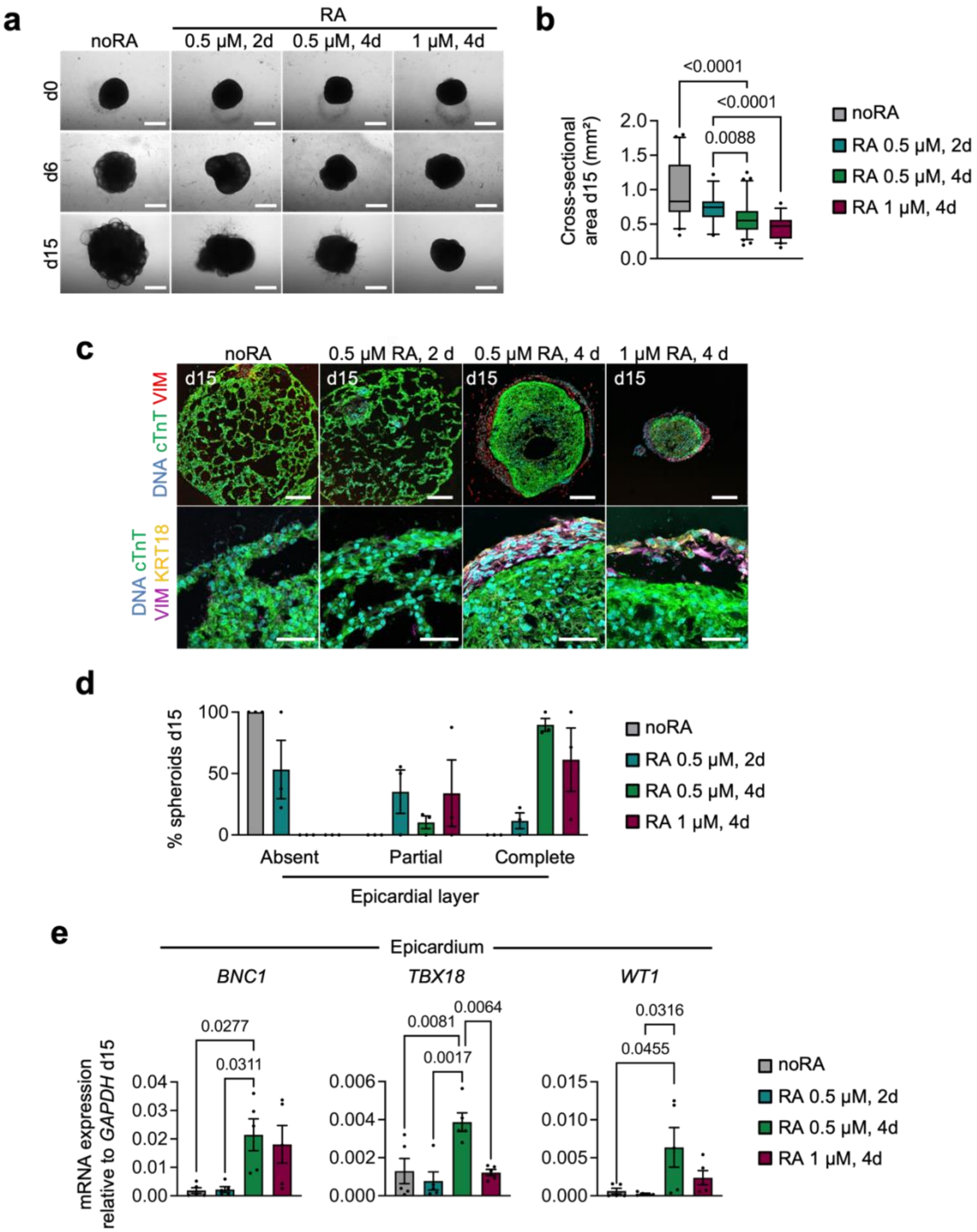

Supplementary Figure 1. Effects of retinoic acid dosage and timing on the formation of epicardial cells during 3D cardiac differentiation. (a) Representative bright field pictures of spheroids at days 0, 6, and 15 of cardiac differentiation without retinoic acid (noRA), with 0.5 µM RA days 2 and 3 (2d) or days 2 to 5 (4d), or with 1 µM RA days 2 to 5 (4d). Scale bars = 500 µm. **(b)** Cross-sectional area of spheroids at day 15 of differentiation without retinoic acid (noRA; N =

43 spheroids), with 0.5 µM RA 2d (N = 33 spheroids), with 0.5 µM RA 4d (N = 70 spheroids), or with 1 µM RA 4d (N = 39 spheroids); 3 independent differentiations per group. Box plots indicate the median, 25th and 75th percentile, with whiskers extending to the 5th and 95th percentiles; Kruskal-Wallis test with Dunn's multiple comparisons test. **(c,d)** Representative images of immunostaining for cardiac troponin T (cTnT) and vimentin (VIM) (top) or cTnT, VIM, and cytokeratin 18 (KRT18) (bottom) at day 15 of differentiation without retinoic acid (noRA), with 0.5 µM RA 2d, with 0.5 µM RA 4d, or with 1 µM RA 4d. Scale bars top = 200 µm, bottom = 50 µm. (c) Corresponding percentage of spheroids with an absent, partial or complete epicardial layer at day 15. Mean ± SEM; N = 3 independent differentiations per group. (d) **(e)** mRNA expression of the epicardial markers *BNC1*, *TBX18*, and *WT1* relative to *GAPDH* at day 15 of differentiation without retinoic acid (noRA), with 0.5 µM RA 2d, with 0.5 µM RA 4d, or with 1 µM RA 4d. Mean ± SEM; N = 5 from 3 independent differentiations per group; one-way ANOVA with Tukey's multiple comparisons test.

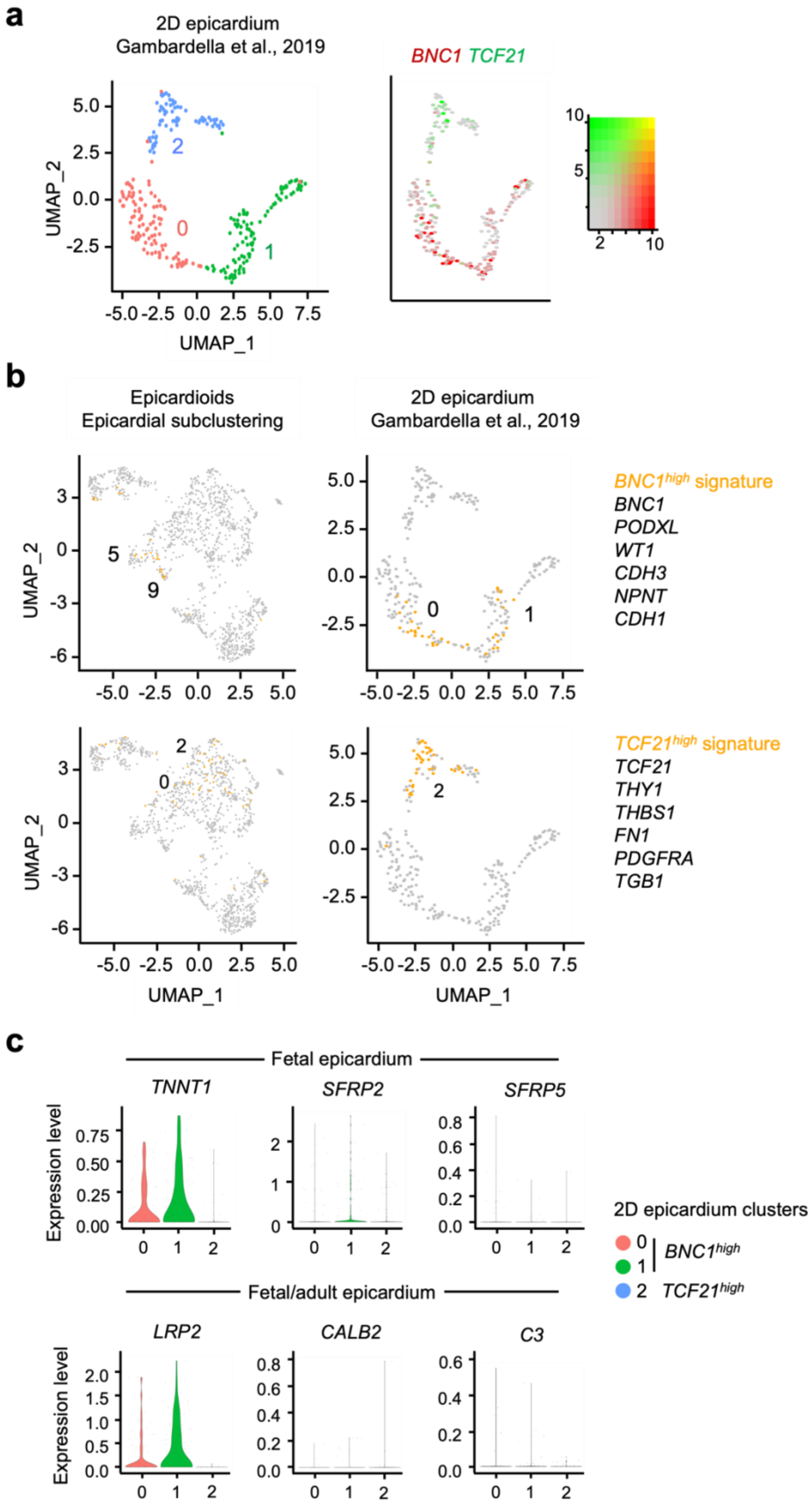

a
2D epicardium
Gambardella et al., 2019
UMAP_2
UMAP_1
BNC1 TCF21
b
Epicardioids
Epicardial subclustering
2D epicardium
Gambardella et al., 2019
BNC1high signature
BNC1
PODXL
WT1
CDH3
NPNT
CDH1
TCF21high signature
TCF21
THY1
THBS1
FN1
PDGFRA
TGB1
c
Fetal epicardium
TNNT1
SFRP2
SFRP5
Expression level
2D epicardium clusters
0
1
BNC1high
2 TCF21high
Fetal/adult epicardium
LRP2
CALB2
C3

Supplementary Figure 2. Comparison of the epicardial populations in epicardioids with epicardial cells obtained in 2D differentiation. (a) (Left) UMAP plot showing the three clusters obtained by reanalyzing the scRNAseq dataset of Gambardella et al. 2019[11], consisting of epicardial cells obtained by directed 2D differentiation of hiPSCs. (Right) The distinction between *BNC1^high^* and *TCF21^high^* populations reported by Gambardella and colleagues is conserved, as shown by exclusive expression of *BNC1* (red) and *TCF21* (green). **(b)** (Top) UMAP plots showing cells co-expressing *BNC1*, *PODXL*, *WT1*, *CDH3*, *NPNT*, and *CDH1* (*BNC1^high^* signature reported by Gambardella et al.) in epicardial cells from epicardioids (left) and in 2D epicardial cells from Gambardella et al., 2019 (right). (Bottom) UMAP plots showing cells co-expressing *TCF21*, *THY1*, *THBS1*, *FN1*, *PDGFRA*, and *TGFB1* (*TCF21^high^* signature reported by Gambardella et al) in epicardial cells from epicardioids (left) and in 2D epicardial cells (right). The main clusters matching the signature in each dataset are annotated. **(c)** Violin plots showing the expression levels of markers of human epicardium in the 2D epicardium clusters from Gambardella et al. *TNNT1*, *SFRP2*, and *SFRP5* are expressed in fetal epicardium; *LRP2*, *CALB2*, and *C3* are shared by fetal and adult epicardium.

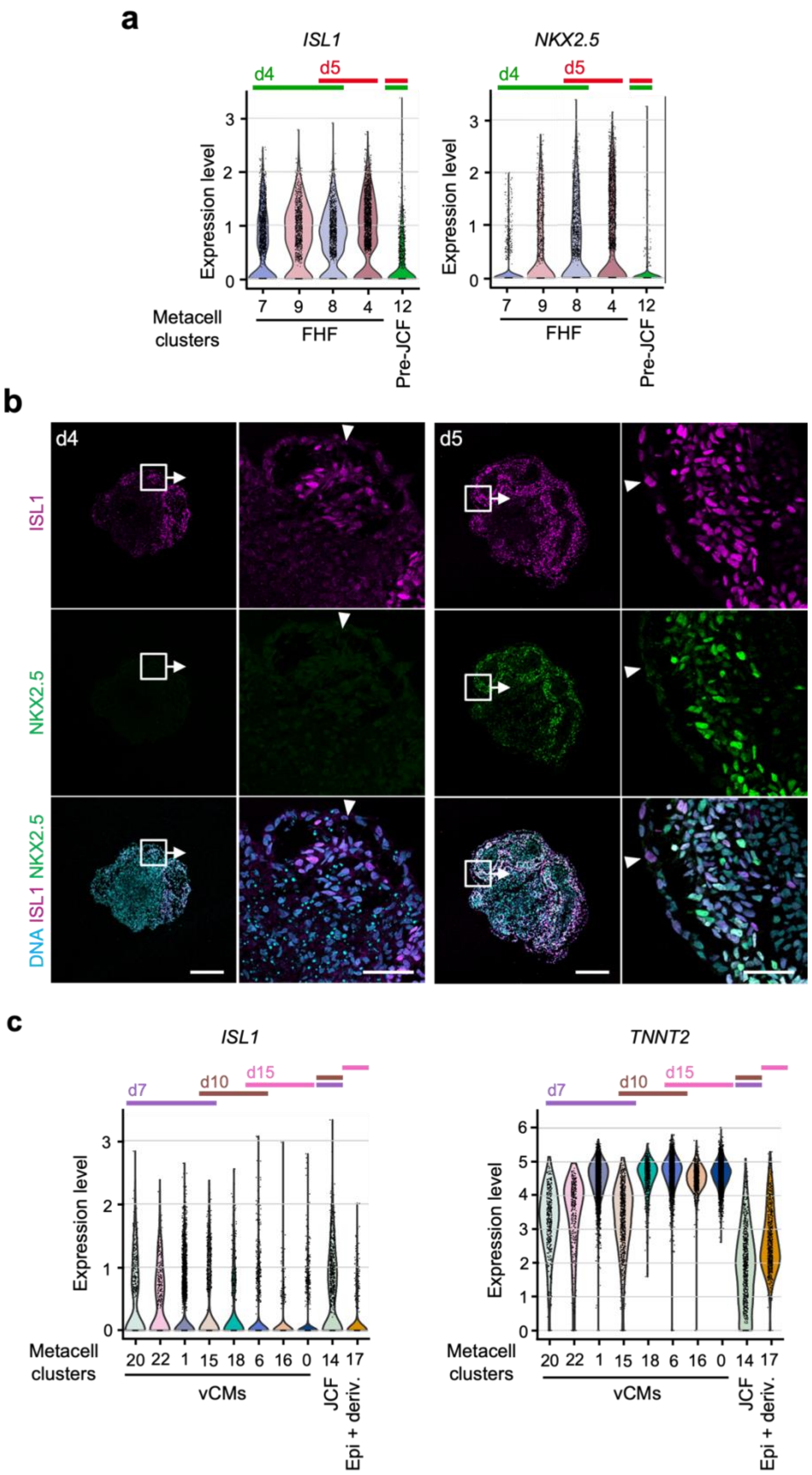

a
ISL1
NKX2.5
d4
d5
Expression level
Metacell clusters
7 9 8 4 12
FHF
Pre-JCF
b
d4
d5
ISL1
NKX2.5
DNA ISL1 NKX2.5
c
ISL1
TNNT2
d7
d10
d15
20 22 1 15 18 6 16 0 14 17
vCMs
JCF
Epi + deriv.

Supplementary Figure 3. ISL1$^+$/NKX2.5$^-$ JCF progenitors and ISL1$^+$/NKX2.5$^+$ FHF progenitors segregate in early epicardioids. (a) Violin plots showing the expression levels of *ISL1* and *NKX2.5* in the indicated FHF and pre-JCF metacells clusters, predominantly present in epicardioids at the indicated days. **(b)** Representative images of immunostaining for ISL1 and NKX2.5 in epicardioids at days 4 (left) and 5 (right). Arrowheads show examples of a layer of ISL1$^+$/NKX2.5$^-$ JCF progenitors segregating from ISL1$^+$/NKX2.5$^+$ FHF progenitors. For each day scale bars left = 200 µm, right = 50 µm. **(c)** Violin plots showing the expression levels of *ISL1* and *TNNT2* in the indicated vCM, JCF and epicardial metacells clusters, predominantly present in epicardioids at the indicated days. FHF: first heart field; JCF: juxta-cardiac field; vCMs: ventricular cardiomyocytes; Epi + deriv.: epicardium and derivatives.

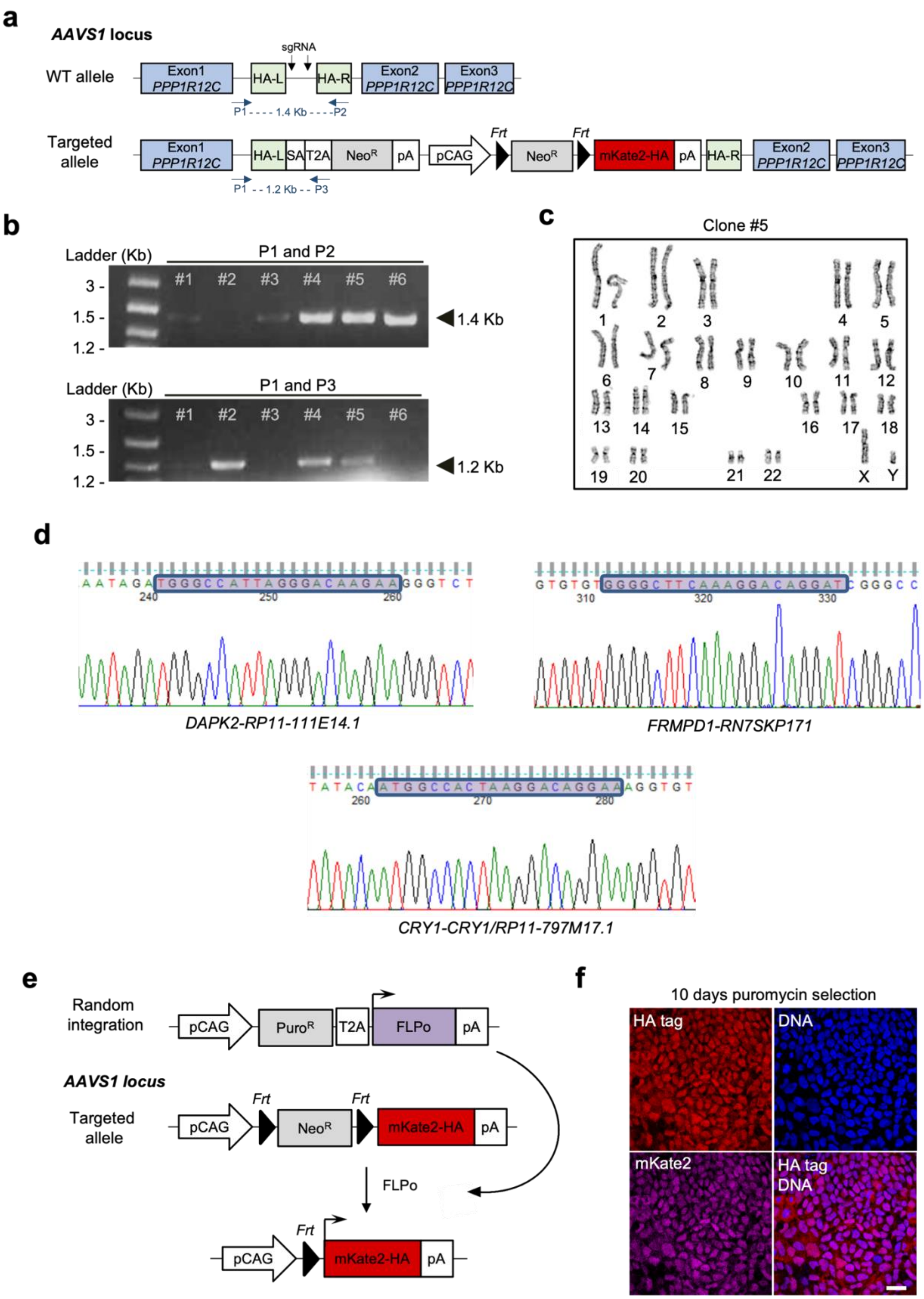
a
AAVS1 locus
WT allele
Exon1 PPP1R12C
HA-L
sgRNA
HA-R
Exon2 PPP1R12C
Exon3 PPP1R12C
P1 - - - - 1.4 Kb - - - -P2
Targeted allele
SA
T2A
NeoR
pA
pCAG
Frt
mKate2-HA
P1 - - 1.2 Kb - - P3
b
Ladder (Kb)
P1 and P2
#1 #2 #3 #4 #5 #6
3 -
1.5 -
1.2 -
1.4 Kb
P1 and P3
1.2 Kb
c
Clone #5
1 2 3 4 5
6 7 8 9 10 11 12
13 14 15 16 17 18
19 20 21 22 X Y
d
AATAGATGGGCCATTAGGGACAAGAAGGGTCT
240 250 260
DAPK2-RP11-111E14.1
GTGTGTGGGGCTTCAAAGGACAGGATCGGGCC
310 320 330
FRMPD1-RN7SKP171
TATACAATGGCCACTAAGGACAGGAAAGGTGT
260 270 280
CRY1-CRY1/RP11-797M17.1
e
Random integration
pCAG
PuroR
T2A
FLPo
pA
AAVS1 locus
Targeted allele
Frt
NeoR
mKate2-HA
FLPo
f
10 days puromycin selection
HA tag
DNA
mKate2
HA tag DNA

Supplementary Figure 4. Generation and validation of the AAVS1-CAG-FRT-flanked STOP-mKate2-HA hiPSC reporter line. (a) Schematic of the *AAVS1* locus targeted with the CAG-FRT-flanked STOP-mKate2-HA construct. Vertical arrows indicate sgRNA targeting sites. Horizontal arrows represent PCR primers for genotyping *AAVS1* locus targeting and homozygosity, respectively. Blue boxes indicate *PPP1R12C* exons; green boxes indicate the regions of homology. **(b)** Representative PCR genotyping of selected hiPSC clones by amplification of the normal (P1 and P2) and targeted alleles (P1 and P3). N = 45 clones total from 2 independent experiments, #5 selected for further experiments. **(c)** Normal karyotype of clone #5 confirmed after CRISPR/Cas9 editing. **(d)** Sanger sequencing of the top 3 predicted off-target sites, confirming no edits at these sites in reporter hiPSCs. **(e,f)** Schematic of the CAG-Puro-2A-FLPo vector used for validation of reporter expression. Puromycin is used to select cells with random integration of the vector; flippase (FLPo) expression mediates the excision of the STOP cassette (FRT-flanked neomycin) in the targeted *AAVS1* locus, allowing mKate2 expression. (e) Immunostaining for the HA-tag and detection of endogenous mKate2 after transfection with the CAG-Puro-2A-FLPo vector and 10 days of puromycin treatment, representative of 2 independent experiments, confirming that the FLP-FRT system works correctly in reporter hiPSCs. Scale bar = 25 μm. (f)

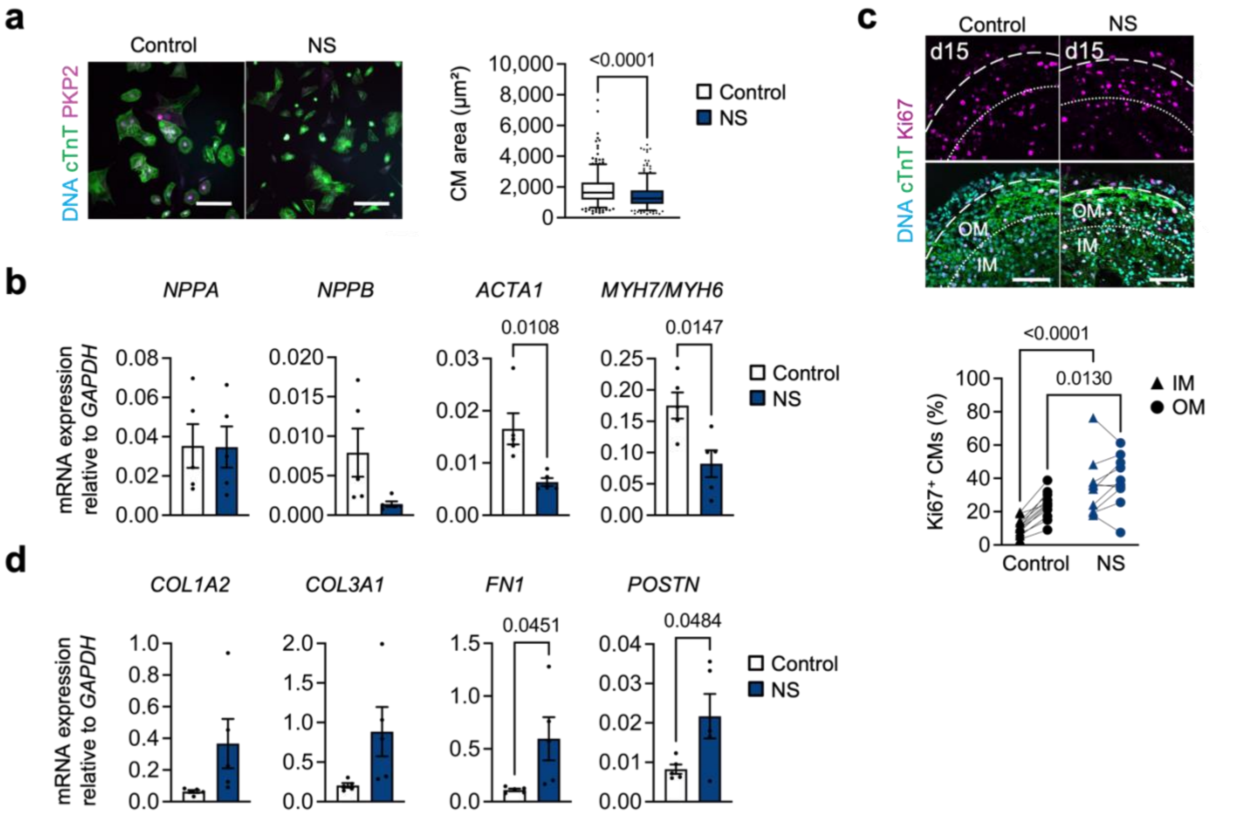

Supplementary Figure 5. Epicardioids from Noonan syndrome patients show myocytic hyperproliferation rather than classical hypertrophic programs. (a) (Left) Representative images of cardiomyocytes (CMs) re-seeded after dissociation of day-30 control or Noonan syndrome (NS) epicardioids and stained for cardiac troponin T (cTnT) and the desmosomal marker plakophilin-2 (PKP2). Scale bars = 100 µm. (Right) Corresponding quantification of CM area, box plots indicate the median, 25th and 75th percentile, with whiskers extending to the 5th and 95th percentiles. Control: N = 379 CMs, NS: N = 360 CMs; 3 differentiations per group; unpaired two-tailed *t*-test. **(b)** mRNA expression of *NPPA*, *NPPB*, *ACTA1*, and *MYH7/MYH6* ratio relative to *GAPDH* in day-30 control or NS epicardioids. Mean ± SEM; N = 5 epicardioids from 3 independent differentiations per group; unpaired two-tailed *t*-test. **(c)** (Top) Representative images of immunostaining for cardiac troponin T (cTnT) and Ki67 in control and NS spheroids at day 15. Hatched lines = outer edge of the myocardium; dotted lines = separation between the outer (OM, 50 µm layer) and inner myocardium (IM). Scale bars = 50 µm. (Bottom) Percentage of Ki67^{+} cardiomyocytes (CMs) in the OM and IM of control and NS epicardioids at day 15. Dot plot showing all data points; lines connect the values for OM and IM within the same sample. Control: N = 12 epicardioids; NS: N = 10 epicardioids; 3 independent differentiations per group; two-way

ANOVA with Tukey's multiple comparisons test. **(d)** mRNA expression of *COL1A2*, *COL3A1*, *FN1*, and *POSTN* relative to *GAPDH* in day-30 control or NS epicardioids. Mean ± SEM; N = 5 epicardioids from 3 independent differentiations per group; unpaired two-tailed *t*-test.

Supplementary Table 7. Sequences of primers used for cloning, genotyping and sequencing of the AAVS1-CAG-Frt-flanked STOP-mKate2HA reporter

**Name**	***Target***	**Primers (5′-3′)**	
Cloning of donor construct	pCAFNF-GFP plasmid	PacI_Sv40-PolyA_Fw	CCTTAATTAACGAAATGACCGACCAAGCGA
Cloning of donor construct	pCAFNF-GFP plasmid	EcoRI_Sv40-PolyA_Rv	CCGGAATTCCGGATTTGATCCAGACATGA
Cloning of donor construct	p3E-mKate2-HA no-pA plasmid	SwaI_mKate2-HA_Fw	GGTACGATTTAAATCATGGTGAGCGAGCTGATTAA
Cloning of donor construct	p3E-mKate2-HA no-pA plasmid	PacI_mKate2-HA_Rv	CCGGTTAATTAATTATGCTGCGTAGTCTGGTACGTCGT
**Name**	***Target***	**Primers (5′-3′)**	
Construct sequencing primers	pAAVS1- CAG- Frt-flanked STOP - mKate2HA-polyA plasmid	AAVS1_mKate2_Fw1	CAAGGGGGAGGATTGGGAAGA
		AAVS1_mKate2_Fw2	TTCCTTTTATGGCGAGGCGG
		AAVS1_mKate2_Fw3	GGGGGTGGCGGCAGGTGGGG
		AAVS1_mKate2_Fw4	GAATTGATTAATTCGAGCGAACGCG
		AAVS1_mKate2_Rv5	AGGAACTTCTCGGATTTGATCCAGA
		AAVS1_mKate2_Fw6	TCTGGATCAAATCCGAGAAGTTCCT
		AAVS1_mKate2_Fw7	GGACACCAGCCTCCAGGACG
		AAVS1_mKate2_Fw8	TTAATTAACGAAATGACCGACC
		AAVS1_mKate2_Rv9	GGTGGCCACTGAGAACCGGG
**Name**	***Target***	**Primers (5′-3′) for PCR screening**	
Genotyping	*AAVS1* locus after CAG- Frt-flanked STOP -mKate2HA-polyA insertion	P1_Fw	TCGACTTCCCCTCTTCCGATG
		P2_Rv	CTCAGGTTCTGGGAGAGGGTAG
		P3_Rv	GAGCCTAGGGCCGGGATTCTC
		**Primers (5′-3′) for PCR amplification**	**Primers (5′-3′) for Sanger sequencing**
		Fw:TCGACTTCCCCTCTTCCGATG<br>Rv: GAGCCTAGGGCCGGGATTCTC	Fw:GGACCACCTTATATTCCCAGGGC<br>Rv:GAGCCTAGGGCCGGGATTCTC<br>Rv:GGCTAAAGCCAGGGAGACGGG
		Fw:GGACCACCTTATATTCCCAGGGC<br>Rv: TAGAAGGCACAGTCGAGG	Fw:GGACCACCTTATATTCCCAGGGC<br>Rv: TAGAAGGCACAGTCGAGG
		Fw: GAATTGATTAATTCGAGCGAACGCG<br>Rv: AGGAACTTCTCGGATTTGATCCAGA	Fw: GAATTGATTAATTCGAGCGAACGCG<br>Fw: CGTTGGCTACCCGTGATATT

			Rv: AGGAACTTCTCGGATTTGATCCAGA<br>Rv: GCCCAGTCATAGCCGAATAG
		Fw: TCTGGATCAAATCCGAGAAGTTCCT<br>Rv: GAATTCCGGATTTGATCCAGACATGA	Fw: TCTGGATCAAATCCGAGAAGTTCCT<br>Fw: GGACACCAGCCTCCAGGACG<br>Fw:TTAATTAACGAAATGACCGACC<br>Rv:TGACGTTGTAGATGAGGCAG
		Fw: CTTAATTAACGAAATGACCGACCAAGCGA<br>Rv: CCAAGTAGGTGGCCTGGGGC	Fw: CTTAATTAACGAAATGACCGACCAAGCGA<br>Rv: CTCAGGTTCTGGGAGAGGGTAG<br>Rv: CCAAGTAGGTGGCCTGGGGC
Off-target analysis	***Target***	**Off target gRNA**	**Primers (5′-3′) PCR amplification and sequencing**
	DAPK2-RP11-111E14.1	TGGGCCATTAGGGACAAGAA	Fw:CACAGAGACATGTCTCTTTGTG<br>Rv:GACAATGGCACATCTGATCCAG
	FRMPD1-RN7SKP171	GGGGCTTCAAAGGACAGGAT	Fw:AGCTCCGGAGATGATCAAGTACAG<br>Rv:AAAGTGCATGCAGTTCCGTAGTG
	CRY1-CRY1/RP11-797M17.1	ATGGCCACTAAGGACAGGAA	Fw:CAGTGCTCTGATCCCTAGAAGGAC<br>Rv:TGTGAATTTAGACGTGGGACTG

Supplementary Table 8. Primary antibodies

Target	Host species	Manufacturer	Catalog number	Concentration
α-SMA	Mouse	Sigma-Aldrich	A2547	1:400 (IF)
CD31	Sheep	R&D	AF806	1:200 (IF)
Collagen III	Rabbit	Invitrogen	PA5-34787	1:100 (IF)
cTnT	Mouse	Invitrogen	MA512960	1:300 (IF)
cTnT	Rabbit	Abcam	ab92546	1:300 (IF)
E-cadherin	Mouse	Abcam	ab1416	1:100 (IF)
Fibronectin	Rabbit	Abcam	ab2413	1:400 (IF)
HA tag	Rabbit	Abcam	ab9110	1:200 (IF)
Ki67	Mouse	BD Biosciences	556003	1:300 (IF)
KRT18	Mouse	Abcam	ab668	1:100 (IF)
IGF1R	Goat	R&D	AF-305	1:100 (IF)
IGF2	Mouse	Invitrogen	MA5-17096	1:100 (IF)
ISL1	Mouse	Developmental Studies Hybridoma bank	39.4D5	1:100 (IF)
NRP2	Goat	R&D	AF2215	1:100 (IF)<br>200, 500 µg/mL (blocking)
NKX2.5	Rabbit	Novus Biologicals	NBP1-31558	1:100 (IF)
PKP2	Guinea pig	Origene	AP09554SU-N	1:100 (IF)
TCF21	Rabbit	Sigma-Aldrich	HPA013189	1:100 (IF)
TBX18	Rabbit	Abcam	ab115262	1:100 (IF)
Vimentin	Chicken	Abcam	ab24525	1:400 (IF)
VE-cadherin	Mouse	Invitrogen	14-1449-82	1:200 (IF)
TJP1	Mouse	Invitrogen	33-9100	1:100 (IF)
Twist1	Sheep	R&D	AF6230	1:50 (IF)

IF: immunofluorescence

Supplementary Table 9. Secondary antibodies

Target species	Host species	Conjugate	Manufacturer	Catalog number	Concentration
Rabbit	Goat	Alexa Fluor 488	Invitrogen	A11008	1:500
Rabbit	Goat	Alexa Fluor 647	Invitrogen	A32733	1:500
Mouse	Goat	Alexa Fluor 594	Invitrogen	A11005	1:500
Mouse	Goat	Alexa Fluor 488	Invitrogen	A11001	1:500
Guinea pig	Goat	Alexa Fluor 594	Invitrogen	A11076	1:500
Chicken	Goat	Alexa Fluor 594	Invitrogen	A11042	1:500
Donkey	Sheep	Alexa Fluor 488	Invitrogen	A11015	1:500

Supplementary Table 10. Conditions of single-cell dissociation of epicardioids

Time point	Number of epicardioids	Dissociation time
Day 2	13	45 min
Day 3	11	45 min
Day 4	10	45 min
Day 5	8	45 min
Day 7	8	50 min
Day 10	6	50 min
Day 15	6	55 min
Day 30	6	60 min

Supplementary Table 11. qPCR primers

Gene	Species	Sequence	
ACTA1	Human	Forward	5’ GTGATCACCATCGGAAATGAA
		Reverse	5’ TCATGATGCTGTTGTAGGTGGT
COL1A2	Human	Forward	5’ CCTGGTGCTAAAGGAGAAAGAGG
		Reverse	5’ ATCACCACGACTTCCAGCAGGA
COL3A1	Human	Forward	5’ TGGTCTGCAAGGAATGCCTGGA
		Reverse	5’ TCTTTCCCTGGGACACCATCAG
GAPDH	Human	Forward	5’ TCCTCTGACTTCAACAGCGA
		Reverse	5’ GGGTCTTACTCCTTGGAGGC
MYH6	Human	Forward	5’ TCAGGATTCTCCGTGAAGGG
		Reverse	5’ CTCTTCCTTGTCATCGGGCA
MYH7	Human	Forward	5’ TGTAGACACACTTGAGTAGCCC
		Reverse	5’ ACGGTCACTGTCTTGCCATA
NPPA	Human	Forward	5’ CAACGCAGACCTGATGGATTT
		Reverse	5’ AGCCCCCGCTTCTTCATTC
NPPB	Human	Forward	5’ CAGCCTCGGACTTGGAAAC
		Reverse	5’ GCTCCAGGGATGTCTGCTC
FN1	Human	Forward	5’ CTGGCCAGTCCTACAACCAG
		Reverse	5’ CGGGAATCTTCTCTGTCAGCC
POSTN	Human	Forward	5’ CCGAGCCTTGTATGTATGTTATG
		Reverse	5’ TTTGTACACCAAGCACCTATTT
