# Supplementary material for: Epicardioid single-cell genomics uncovers principles of human epicardium biology in heart development and disease
Source: Nat Biotechnol. 2023 Apr 3;41(12):1787–800. doi: 10.1038/s41587-023-01718-7 (PMC10713454; doi:10.1038/s41587-023-01718-7)
Supplement: Supplementary file 2 — Reporting Summary [file 41587_2023_1718_MOESM2_ESM.pdf]

nature research

Corresponding author(s): Alessandra Moretti

Last updated by author(s): Feb 13, 2023

Reporting Summary

Nature Research wishes to improve the reproducibility of the work that we publish. This form provides structure for consistency and transparency in reporting. For further information on Nature Research policies, see our Editorial Policies and the Editorial Policy Checklist.

Statistics

For all statistical analyses, confirm that the following items are present in the figure legend, table legend, main text, or Methods section.

n/a	Confirmed	
☐	☒	The exact sample size (*n*) for each experimental group/condition, given as a discrete number and unit of measurement
☐	☒	A statement on whether measurements were taken from distinct samples or whether the same sample was measured repeatedly
☐	☒	The statistical test(s) used AND whether they are one- or two-sided<br>*Only common tests should be described solely by name; describe more complex techniques in the Methods section.*
☒	☐	A description of all covariates tested
☐	☒	A description of any assumptions or corrections, such as tests of normality and adjustment for multiple comparisons
☐	☒	A full description of the statistical parameters including central tendency (e.g. means) or other basic estimates (e.g. regression coefficient) AND variation (e.g. standard deviation) or associated estimates of uncertainty (e.g. confidence intervals)
☐	☒	For null hypothesis testing, the test statistic (e.g. F, t, r) with confidence intervals, effect sizes, degrees of freedom and P value noted<br>*Give P values as exact values whenever suitable.*
☒	☐	For Bayesian analysis, information on the choice of priors and Markov chain Monte Carlo settings
☒	☐	For hierarchical and complex designs, identification of the appropriate level for tests and full reporting of outcomes
☐	☒	Estimates of effect sizes (e.g. Cohen's *d*, Pearson's *r*), indicating how they were calculated

Our web collection on statistics for biologists contains articles on many of the points above.

Software and code

Policy information about availability of computer code

Data collection	Leica application suite X v3.5.7.23225<br>Andor Solis V4.30.30034.0
Data analysis	All software used for the data analysis is described in detail in the methods section of the manuscript. The following software packages including version numbers were used:<br>GraphPad Prism v9.1.0.; ImageJ v1.53a; MATLAB vR2021a; Tophat v2.1.1; Bowtie v2.4.5; MACS v2.2.7.1; cellranger pipeline v6.1.1; Rstudio v1.1.453; R v3.5.1, v4.1.3; Seurat R package v4.0.1, v4.1.1; clustree R package v0.4.3; pheatmap R package v1.0.12; yaGST R package v2017.08.25; clusterProfiler R package v2.15.1; Cell Ranger ATAC v1.2.0; Signac R package v1.7.0; CLI of the python implementation CellPhoneDB v2.1.7; AMULET v1.1; GLUE v0.2.3; CellRank v1.5.1; scanpy v1.9.1; python implementation of magic v3.0.0; scVelo v0.2.4; Pando R package v1.0.1; liftOver R package v1.18.0; scikit-learn v1.1.1.<br><br>Jupyter notebooks to reproduce the metacell analysis and figures are available at https://github.com/theislab/epicardiods_analysis.

For manuscripts utilizing custom algorithms or software that are central to the research but not yet described in published literature, software must be made available to editors and reviewers. We strongly encourage code deposition in a community repository (e.g. GitHub). See the Nature Research guidelines for submitting code & software for further information.

Data

Policy information about availability of data

All manuscripts must include a data availability statement. This statement should provide the following information, where applicable:

- Accession codes, unique identifiers, or web links for publicly available datasets
- A list of figures that have associated raw data
- A description of any restrictions on data availability

All scRNA-seq and scATAC-seq data that support the findings of this study can be found at Gene Expression Omnibus under the accession number GSE196516. Reads containing sequence information were aligned using the GRCh37 reference genome and ENSEMBL gene annotation (http://igenomes.illumina.com.s3-website-us-east-1.amazonaws.com/Homo_sapiens/Ensembl/GRCh37/Homo_sapiens_Ensembl_GRCh37.tar.gz).
For analysis of the 2D epicardium scRNA-seq dataset from Gambardella et al. (GSE122827), we downloaded the raw data from https://www.ncbi.nlm.nih.gov/geo/query/acc.cgi?acc=GSE122827. We downloaded the UMI counts of the Cui et al. dataset (GSE06118) from https://www.ncbi.nlm.nih.gov/geo/query/acc.cgi?acc=GSE106118. Source data for all figures are provided with this paper. Any other data supporting the findings of this study are available from the corresponding author on reasonable request.

Field-specific reporting

Please select the one below that is the best fit for your research. If you are not sure, read the appropriate sections before making your selection.

☒ Life sciences ☐ Behavioural & social sciences ☐ Ecological, evolutionary & environmental sciences

For a reference copy of the document with all sections, see nature.com/documents/nr-reporting-summary-flat.pdf

Life sciences study design

All studies must disclose on these points even when the disclosure is negative.

Sample size	Sample size was not pre-determined. Based on the high robustness of epicardioid differentiation as shown in the manuscript, we considered a sample size of at least n = 3 to be sufficient for experiments containing statistical analysis.
Data exclusions	Spheroids showing failed cardiac differentiation (no spontaneous beating) were excluded from analysis, except when determining the efficiency of epicardioid formation (presented in Extended Data Fig. 1g). No other data were excluded.
Replication	The efficiency of epicardioid formation was quantified in 3 hiPSC lines and 1 hESC line, which showed high reproducibility of the protocol. With the exception of scRNA-seq/ATAC-seq analysis, experiments were conducted at least 3 times independently, and were based on at least 3 independent differentiations (rarely: 2 independent experiments, as indicated). All attempts for replication were successful.
Randomization	Spheroids were randomly assigned to experimental groups receiving different treatments (noRA vs different RA dosages, Linsitinib vs control, IGF2 vs control, NRP2 ab vs control, endothelin-1 vs control). Randomization was not relevant to other experiments where no comparisons were performed within the same batch of differentiation.
Blinding	Investigators were not blinded to experimental groups. For almost all experiments relying on the quantification of immunofluorescence images, blinding was not feasible since different conditions were identifiable based on morphological characteristics. For the analysis of the qPCR data, blinding was not necessary since this analysis is observer-independent.

Reporting for specific materials, systems and methods

We require information from authors about some types of materials, experimental systems and methods used in many studies. Here, indicate whether each material, system or method listed is relevant to your study. If you are not sure if a list item applies to your research, read the appropriate section before selecting a response.

Materials & experimental systems

n/a	Involved in the study	
☐	☒	Antibodies
☐	☒	Eukaryotic cell lines
☒	☐	Palaeontology and archaeology
☒	☐	Animals and other organisms
☐	☒	Human research participants
☒	☐	Clinical data
☒	☐	Dual use research of concern

Methods

n/a	Involved in the study	
☒	☐	ChIP-seq
☒	☐	Flow cytometry
☒	☐	MRI-based neuroimaging

Antibodies

Antibodies used	Primary antibodies<br>anti-alpha-smooth muscle actin, clone 1A4, Sigma-Aldrich #A2547<br>anti-CD31, polyclonal, R&D #AF806<br>anti-collagen III, polyclonal, Invitrogen #PA5-34787<br>anti-cardiac troponin T, clone 13-11, Invitrogen #MA512960<br>anti-cardiac troponin T, clone EPR3696, abcam #ab92546<br>anti-fibronectin, polyclonal, abcam #ab2413<br>anti-Ki67, clone B56, BD Biosciences #556003<br>anti-KRT18, clone C-04, abcam #ab668<br>anti-ISL1, clone 39.4D5, Developmental Studies Hybridoma bank #39.4D5<br>anti-NKX2.5, polyclonal, Novus Biologicals #NBP1-31558<br>anti-PKP2, polyclonal, Origene #AP09554SU-N<br>anti-TCF21, polyclonal, Sigma-Aldrich #HPA013189<br>anti-TBX18, polyclonal. abcam #ab115262<br>anti-vimentin, polyclonal, abcam #ab24525<br>anti-VE-cadherin, clone 16B1, Invitrogen #14-1449-82<br>anti-TJP1, clone ZO1-1A12, Invitrogen #33-9100<br>anti-HA-tag, polyclonal, abcam #ab9110<br>anti-NRP2, polyclonal, R&D #AF2215<br>anti-Twist1, polyclonal, R&D #AF6230<br>anti-IGF2, clone 8H1, Invitrogen MA5-17096<br>anti-IGF1R, polyclonal, R&D #AF-305<br>anti-E-cadherin, clone HECD-1, abcam #ab1416<br><br>Secondary antibodies<br>Goat anti-rabbit Alexa Fluor 488, Invitrogen #A11008<br>Goat anti-rabbit Alexa Fluor 647, Invitrogen #A32733<br>Goat anti-mouse Alexa Fluor 594, Invitrogen #A11005<br>Goat anti-mouse Alexa Fluor 488, Invitrogen #A11001<br>Goat anti-guinea pig Alexa Fluor 594, Invitrogen #A11076<br>Goat anti-chicken Alex Fluor 594, Invitrogen #A11042<br>Donkey anti-sheep Alexa Fluor 488. Invitrogen #A11015
Validation	All primary antibodies were validated for immunocytochemistry/immunohistochemistry analysis of human samples by the respective manufacturers. All secondary antibodies were validated for immunocytochemistry/immunohistochemistry by the respective manufacturers.

Eukaryotic cell lines

Policy information about cell lines

Cell line source(s)	All hiPSC lines were generated from individuals recruited at the Technical University of Munich (Germany). Two hiPSC lines were generated from healthy donors, as previously described (MRIi003-A: Moretti et al. 2020, Nature Medicine; MRIi001-A: Krane et al. 2021, Circulation). The hiPSC line MRIi025-A was generated from a patient with Noonan syndrome, as previously described (Meier et al. 2022, iScience). The hiPSC line MRIi003-A-6 (AAVS1-CAG-VSFP) was derived from MRIi003-A as previously described (Zhang et al. 2022, Stem Cell Research). The hiPSC line MRIi003-A-9 (AAVS1-CAG-FRT-flanked STOP-mKate2-HA) was derived from MRIi003-A in this study.<br>The HES-3 (ESIBI3003-A) line was generated by ES Cell International Pte Ltd, Singapore and generously provided by Dr. David A. Elliott of the Murdoch Children's Research Institute and Monash Immunology and Stem Cell Laboratories, Monash University, Clayton, Victoria, Australia.<br>HEK293T cells were purchased from ATCC.
Authentication	The pluripotency, trilineage potential, and karyotype of hPSC lines were validated in this and previous studies (Moretti et al. 2020, Nature Medicine; Krane et al. 2021, Circulation; Zhang et al. 2022, Stem Cell Research; Meier et al. 2022, iScience; International Stem Cell Initiative et al. 2007, Nature Biotechnology; Mallon et al. 2014, Stem Cell Research). Pluripotency was confirmed based on typical colony morphology and the expression of pluripotency markers (determined by immunofluorescence, RT-PCR, and/or flow cytometry). Trilineage potential was assessed by qPCR analysis of specific markers of the three germ layers following trilineage differentiation in embryoid bodies or monolayers. Correct reporting of the AAVS1-CAG-FRT-flanked STOP-mKate2-HA hiPSC line was validated by transfection with a plasmid encoding flippase.
Mycoplasma contamination	All cell lines used tested negative for mycoplasma.
Commonly misidentified lines<br>(See ICLAC register)	No commonly misidentified lines were used.

Human research participants

Policy information about studies involving human research participants

Population characteristics	Human embryonic and fetal hearts obtained from abortion materials between four and twelve weeks of gestation from

Population characteristics	healthy subjects.
Recruitment	Material collection was carried out at Karolinska University Hospital in Huddinge (Sweden). Only after the patient decided to undergo abortion for any reason, the medical staff at the Gynecology department informed her and her partner (or closest relatives) about the possibility to donate the embryo/fetus for research purposes, with documents describing the kinds of research that would be performed and so on. After giving their informed consent for donation of the embryo or fetus, the patient underwent surgical abortion, and the aborted material was dissected under sterile conditions.
Ethics oversight	Karolinska Institutet (Sweden) with the approved ethical permission number (Dnr 2015/1369-31/2).

Note that full information on the approval of the study protocol must also be provided in the manuscript.
