# Supplementary figures and images for: Epicardioid single-cell genomics uncovers principles of human epicardium biology in heart development and disease
Source: Nat Biotechnol. 2023 Apr 3;41(12):1787–800. doi: 10.1038/s41587-023-01718-7 (PMC10713454; doi:10.1038/s41587-023-01718-7)

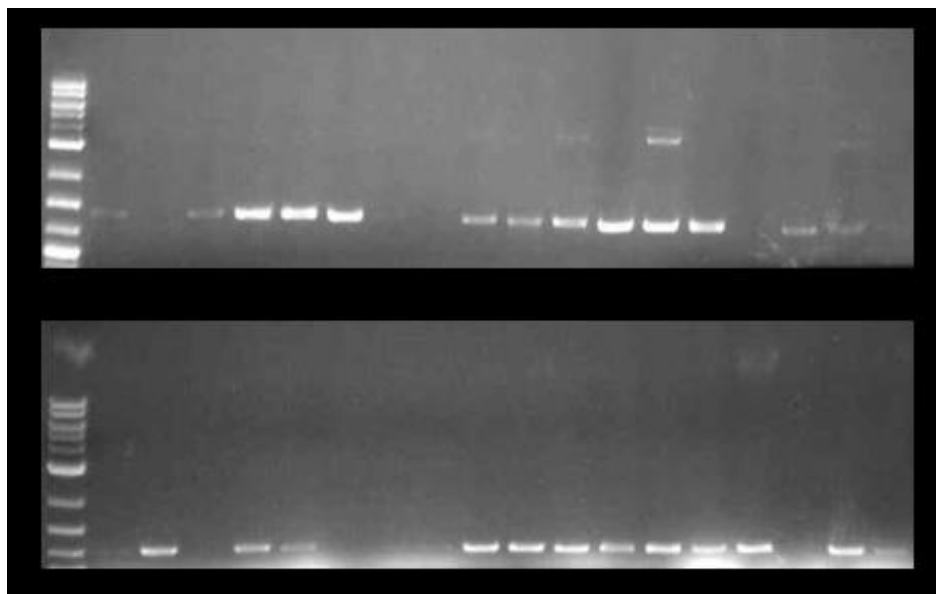

Uncropped scans of the PCR gels presented in Supplementary Figure 4b.

Supplement: Supplementary file 8 — Uncropped scans of the PCR gels presented in Supplementary Fig. 4b. [file 41587_2023_1718_MOESM8_ESM.pdf]
